# Advances in the synthesis of functionalised pyrrolotetrathiafulvalenes

**DOI:** 10.3762/bjoc.11.125

**Published:** 2015-07-03

**Authors:** Luke J O’Driscoll, Sissel S Andersen, Marta V Solano, Dan Bendixen, Morten Jensen, Troels Duedal, Jess Lycoops, Cornelia van der Pol, Rebecca E Sørensen, Karina R Larsen, Kenneth Myntman, Christian Henriksen, Stinne W Hansen, Jan O Jeppesen

**Affiliations:** 1 Department of Physics, Chemistry and Pharmacy, University of Southern Denmark, Campusvej 55, DK-5230, Odense M, Denmark

**Keywords:** heterocycles, protecting groups, sulfur chemistry, tetrathiafulvalene, Ullman coupling

## Abstract

The electron-donor and unique redox properties of the tetrathiafulvalene (TTF, **1**) moiety have led to diverse applications in many areas of chemistry. Monopyrrolotetrathiafulvalenes (MPTTFs, **4**) and bispyrrolotetrathiafulvalenes (BPTTFs, **5**) are useful structural motifs and have found widespread use in fields such as supramolecular chemistry and molecular electronics. Protocols enabling the synthesis of functionalised MPTTFs and BPTTFs are therefore of broad interest. Herein, we present the synthesis of a range of functionalised MPTTF and BPTTF species. Firstly, the large-scale preparation of the precursor species *N*-tosyl-(1,3)-dithiolo[4,5-*c*]pyrrole-2-one (**6**) is described, as well as the synthesis of the analogue *N*-tosyl-4,6-dimethyl-(1,3)-dithiolo[4,5-*c*]pyrrole-2-one (**7**). Thereafter, we show how **6** and **7** can be used to prepare BPTTFs using homocoupling reactions and functionalised MPTTFs using cross-coupling reactions with a variety of 1,3-dithiole-2-thiones (**19**). Subsequently, the incorporation of more complex functionality is discussed. We show how the 2-cyanoethyl protecting group can be used to afford MPTTFs functionalised with thioethers, exemplified by a series of ethylene glycol derivatives. Additionally, the merits of 1,8-diazabicyclo[5.4.0]undec-7-ene (DBU) as an alternative to the most common deprotecting agent, CsOH·H_2_O are discussed. Finally, we show how a copper-mediated Ullman-type reaction can be applied to the *N*-arylation of MPTTFs and BPTTFs using a variety of aryl halides.

## Introduction

Tetrathiafulvalene (TTF) derivatives are of considerable interest in the fields of supramolecular chemistry and molecular machines [1–5], molecular and organic electronics [5–7], chemosensors [1,8–11], coordination chemistry [12–14], catalysis [15] and beyond [16–21]. This owes much to the strong electron-donor character of the TTF moiety and its derivatives, which have been utilized in the formation of charge-transfer (CT) complexes for more than 40 years [21–23].

**Figure 1:**
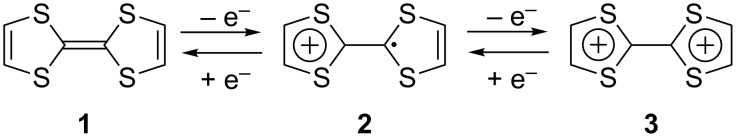
The sequential, reversible oxidation of TTF (**1**) to its stable radical cation (**2**) and dication (**3**) states.

TTF (**1**) (Figure 1) is not aromatic according to the Hückel definition as its 14 π-electrons lack cyclic conjugation. Upon oxidation to the radical cation (**2**) and dication (**3**) states, a gain in aromaticity occurs: **2** contains a single aromatic, 6 π-electron 1,3-dithiolium system, and **3** possesses two such systems (Figure 1). These oxidations occur at low potential (*E*^1^_1/2_ = 0.34 V and *E*^2^_1/2_ = 0.73 V vs Ag/AgCl in MeCN [4]) and can be performed sequentially and reversibly. Additionally, both **2** and **3** are thermodynamically stable. These properties are responsible for the strong electron-donor character of TTF and its derivatives. Furthermore, the precise oxidation potential of a TTF derivative can be changed by the addition of electron-donating or electron-withdrawing substituents [4]. Usually, each of the three stable oxidation states possesses a distinct UV–vis absorption spectrum [4], facilitating studies of redox behaviour.

To exploit the properties of TTF in more complex systems, various functionalised derivatives have been prepared, including both alkylated, arylated and annelated species [7]. A common complication encountered with such functionalised TTFs is the existence of *cis* and *trans* stereoisomers. The investigation of the properties of a single isomer is challenging, not only due to difficulties in the separation of the isomers, but also because it is possible for the isomers to interconvert in the presence of acid or light [24]. Studies of functionalised TTFs must therefore often use mixtures of isomers, although in some cases only one isomer exhibits the desired behaviour [9]. Isomeric mixtures can also complicate the interpretation of spectroscopic data.

**Figure 2:**
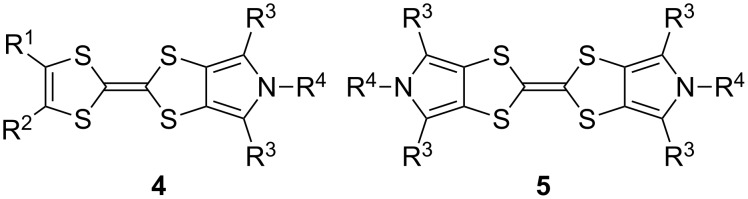
Structures and possible substitution positions of MPTTFs (**4**) and BPTTFs (**5**).

These drawbacks led the group of Jeppesen and Becher to develop pyrrole-annelated TTF derivatives: monopyrrolotetrathiafulvalenes (MPTTFs, **4**) and bispyrrolotetrathiafulvalenes (BPTTFs, **5**) (Figure 2) [4,25]. The presence of either one or two fused pyrrole rings, respectively, eliminates *cis*–*trans* isomerism whilst still allowing for further functionalisation. Methodologies have been developed which facilitate the preparation of MPTTFs and BPTTFs independently substituted in almost all of the positions indicated in Figure 2. This ranges from the addition of simple alkyl, acyl or aryl substituents [13,19,26–28] to the preparation of fused ring systems [11,27], to the incorporation of MPTTFs and BPTTFs into more complex molecular architectures such as macrocycles [8–9,14], calix-pyrroles [1,10–11], calixarenes [29] and porphyrins [30]. Note that for MPTTFs, R^1^ and R^2^ can be either the same or different.

Here, we present recent developments in the synthesis of functionalised MPTTFs and BPTTFs. We report a more convenient and larger scale (>20 g) synthesis of the key building block *N*-tosyl-(1,3)-dithiolo[4,5-*c*]pyrrole-2-one (**6**), than that previously published [25], in addition to the synthesis of its dimethylated analogue, *N*-tosyl-4,6-dimethyl-(1,3)-dithiolo[4,5-*c*]pyrrole-2-one (**7**). We then provide a range of examples where **6** and **7** are used in the preparation of functionalised MPTTFs and BPTTFs. We expand on this by discussing subsequent additional functionalisation of MPTTFs and BPTTFs by two different methods: (i) the use of 2-cyanoethyl-protected thiols as a means to further functionalise MPTTFs with thioethers and (ii) copper-mediated *N*-arylation of both MPTTFs and BPTTFs.

## Results and Discussion

### An improved large-scale synthesis of *N*-tosyl-(1,3)-dithiolo[4,5-*c*]pyrrole-2-one (**6**)

**Scheme 1:**
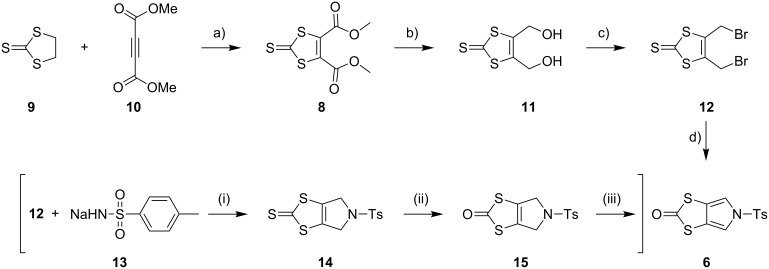
Large-scale synthesis of **6**. Reagents and conditions: a) PhMe, reflux, 19 h, 74%; b) LiBr, NaBH_4_, THF, MeOH, −10 °C → rt, 20 h, 77%; c) PBr_3_, THF, 0 °C → rt, 20 h, 75%; d) (i) **13**, MeCN, DMF, 80 °C, 15 min, (ii) Hg(OAc)_2_, CHCl_3_, AcOH, rt, 24 h, (iii) DDQ, PhCl, reflux, 4 h, 52% (from **12**).

The known compound **6** [4,25] is an important building block in the preparation of MPTTFs and BPTTFs. We have further developed the synthesis previously reported by our group [4,25] and can now isolate **6** in quantities in excess of 20 g. Our current large-scale synthetic strategy (Scheme 1) is comparable to that described in 2000 [25] but requires fewer intermediate purification steps. Diester **8** can be prepared from commercially available ethylene trithiocarbonate (**9**) and dimethyl acetylenedicarboxylate (**10**) at around 100 g scale in 74% yield (based on previously reported large-scale syntheses [31–32]). We have scaled up the reduction of **8** using sodium borohydride and lithium bromide to 40 g scale, obtaining diol **11** with only a small reduction in yield (77% vs 85% at 15 g scale [33]). Compound **11** is then treated with phosphorus tribromide to afford dibromide **12**. The scale up of this reaction to 36 g scale also results in a lower, but still acceptable yield (75% vs 91% at approximately 5 g scale [25]). The conversion of **12** to **6** is achieved in three steps with minimal intermediate purification, beginning with up to 44 g of **12**. Reaction of **12** with sodium tosylamide (**13**) (prepared from tosylamide according to a literature procedure [34]) affords the cyclised product **14**. Crude material of sufficient purity for the following step can be isolated by precipitation (see the Experimental section in the Supporting Information File 1). Transchalcogenation of crude **14** affords **15** and aromatisation of crude **15** using 2,3-dichloro-5,6-dicyano-1,4-benzoquinone (DDQ) gives **6**, which is purified by column chromatography, in 52% overall yield (22.5 g) from **12**. We have also consistently obtained comparable yields of around 55% using the same method at approximately half this scale. The ability to isolate multigram quantities of **6**, which can be stored for years on the shelf, makes the subsequent synthesis of various MPTTF and BPTTF derivatives much more convenient and accessible.

### Synthesis of *N*-tosyl-4,6-dimethyl-(1,3)-dithiolo[4,5-*c*]pyrrole-2-one (**7**)

**Scheme 2:**
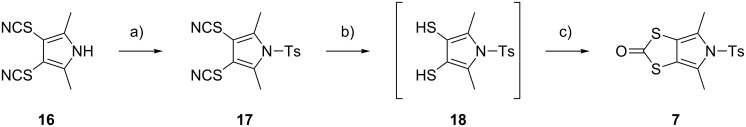
Preparation of **7**. Reagents and conditions: a) TsCl, Et_3_N, DMAP, MeCN, rt → reflux, 3.5 h, 82%; b) (i) LiAlH_4_, THF, 0 °C, 2.5 h, (ii) AcOH, Et_2_O, 0 °C; c) (Im)_2_CO, THF, 0 °C, 1 h, 83% (from **17**).

To the best of our knowledge, the synthesis of **7**, the dimethylated analogue of **6**, has not been previously reported by other groups. *N*-Phenylated and *N*-Boc-protected analogues were, however, reported in 1996 [35]. Based on that work, we have developed a preparation of **7** (Scheme 2) [36]. Functionalised pyrrole **16** was prepared according to the literature [35,37] then tosylated in high yield to afford **17**. The reduction of the thiocyanate moieties with LiAlH_4_ afforded the air-sensitive intermediate **18** (not characterised), which was treated with 1,1’-carbonyldiimidazole to afford **7**, in 83% yield over the two steps.

### Preparation of functionalised MPTTFs and BPTTFs

#### Coupling reactions

**Scheme 3:**
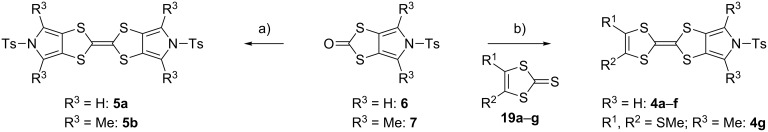
Homo and cross-coupling reactions of **6** or **7** afford BPTTFs and MPTTFs, respectively. Reagents and conditions: a) (EtO)_3_P, 120–130 °C, 5–5.5 h, 76–79%; b) (EtO)_3_P, 120–135 °C, 1–4 h, 70–87%.

Pyrrole-annelated TTF derivatives can be prepared from **6** and **7** by means of coupling reactions in triethyl phosphite (Scheme 3). The known homocoupling reaction of **6** affords the bis-tosylated BPTTF **5a** in high yield with minimal purification [25]. We have obtained a comparable yield (76% vs 84%) working at twice the previously published scale [25]. The equivalent reaction can be conducted using **7** to give BPTTF **5b** with a similar yield of 79% [36], a modest improvement on the reported yield of 73% for the Boc-protected analogue [35].

The synthesis of MPTTFs can be achieved using cross-coupling reactions between **6** and 1,3-dithiole-2-thiones **19** [38]. These reactions (Scheme 3 and Table 1) use an excess of **19** to minimise the formation of **5a** as a byproduct, making it possible to isolate tosylated MPTTFs (such as **4a**–**f**) in high yields. This is believed to be due to the higher reactivity of sulfur ylide intermediates (formed from 1,3-dithiole-2-thiones) with 1,3-dithiole-2-ones than with excess 1,3-dithiole-2-thiones [39]. Table 1 lists a selection of *N*-tosylated MPTTFs **4a**–**f** prepared from **6** and **19a**–**f**, respectively, with yields ranging from 70–87% despite the concomitant formation of homocoupled byproducts. Compound **7** (and related species [27]) can also be used in cross-coupling reactions, exemplified by its reaction with **19g** to give **4g** (Scheme 3 and Table 1) [36]. Our group has previously shown that it is also possible to introduce substituents directly onto the pyrrole ring of MPTTF **4d** [40].

**Table 1:** Cross-coupling reactions between 1,3-dithiole-2-ones and 1,3-dithiole-2-thiones affording MPTTFs.

1,3-Dithiole-2-one	1,3-Dithiole-2-thione	Product	% Yield

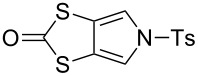 **6**	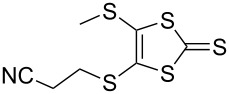 **19a**	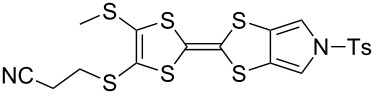 **4a**	85 (lit. 64 [41])
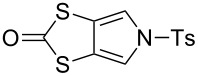 **6**	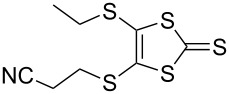 **19b**	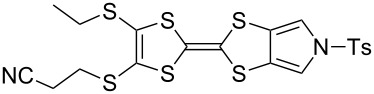 **4b**	83 (lit. 74 [42])
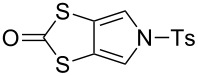 **6**	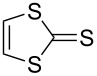 **19c**	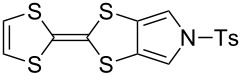 **4c**	71 (lit. 60 [28])
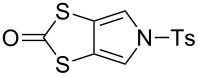 **6**	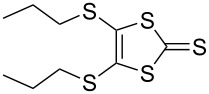 **19d**	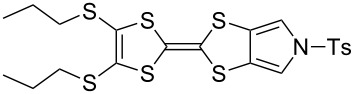 **4d**	87 (lit. 62 [43])
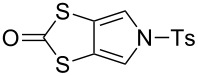 **6**	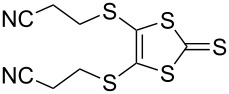 **19e**	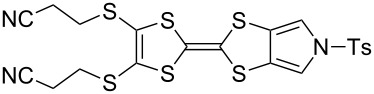 **4e**	70 (lit. 64 [25])
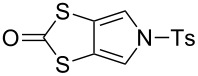 **6**	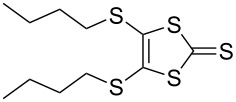 **19f**	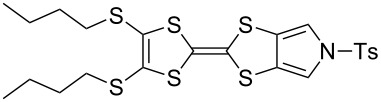 **4f**	85 [44]
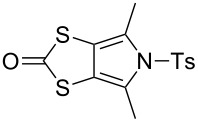 **7**	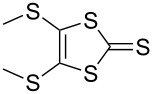 **19g**	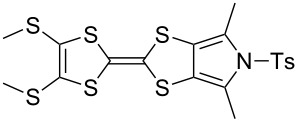 **4g**	80 [36]

#### Further functionalisation of MPTTFs bearing a 2-cyanoethyl-protected thiol

The 2-cyanoethyl protecting group offers a convenient means of synthesising MPTTFs where one or both of R^1^ and R^2^ are thioethers. When only simple alkyl thioethers are targeted, it is often more effective to incorporate these moieties prior to the cross-coupling step that is used to form the TTF core, as described in the previous section. However, many functionalities or larger molecular architectures do not tolerate the harsh coupling conditions and must be added subsequently. The preparation of a series of analogous compounds can also be facilitated by preparing a common, protected MPTTF intermediate, such as **4a**, **4b** or **4e**, in large quantities, particularly in light of the good stability of the cyanoethyl and tosyl protecting groups.

**Scheme 4:**
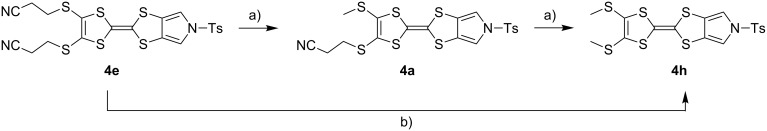
Deprotection and methylation of cyanoethyl-protected thiol moieties on MPTTFs as reported by Jeppesen et al. [25]. Reagents and conditions: a) (i) 1 equiv CsOH·H_2_O, MeOH, THF, rt, 1 h, (ii) MeI, THF, rt, 30 min; b) (i) 1 equiv CsOH·H_2_O, MeOH, THF, rt, 1 h, (ii) MeI, THF, rt, 45 min, (iii) 1 equiv CsOH·H_2_O, MeOH, THF, rt, 1 h, (iv) MeI, THF, rt, 30 min.

As a simple example of this protocol, it has previously been shown that caesium hydroxide monohydrate (CsOH·H_2_O) and methyl iodide can be used to accomplish the transformation of 2-cyanoethyl thioethers to methyl thioethers in high yield for both TTFs [38] and MPTTFs [25]. Thus deprotection and alkylation are achieved in a single synthetic step (e.g. the preparation of **4h** from **4a** in Scheme 4). Furthermore, when R^1^ and R^2^ are both 2-cyanoethyl thioethers (**4e**) these reagents can be used to selectively deprotect and alkylate only one of the two thiols, affording MPTTF **4a** (Scheme 4) [25]. Indeed, the direct preparation of **4h** from **4e** requires two iterative additions of base and alkylating agent. A wide range of other, more complex alkylating agents can be successfully used in place of methyl iodide. For example, functionalised ethylene glycol oligomers have been used in the preparation of rotaxanes and pseudorotaxanes [28,41–42,45].

Although CsOH·H_2_O is most commonly used, other bases are also known to remove the cyanoethyl protecting group from TTF derivatives [46]. However, some of these bases (e.g. sodium methoxide) are incompatible with the *N*-tosyl protecting group typically present during the synthesis of functionalised MPTTFs (see Scheme 3), and therefore cannot be used with these materials.

**Scheme 5:**
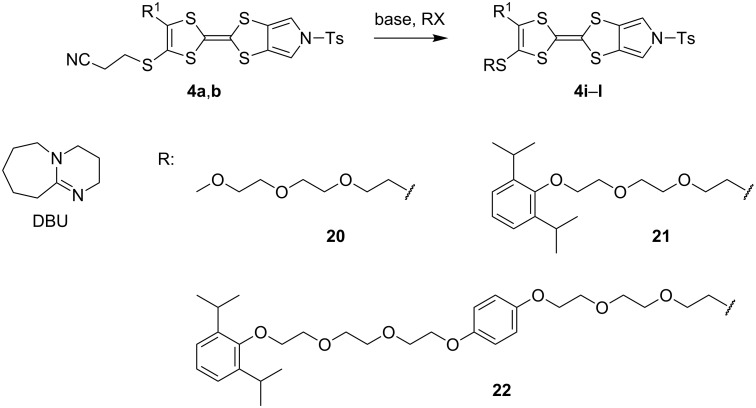
Deprotection and alkylation of cyanoethyl-protected thiol moieties on MPTTFs using CsOH·H_2_O or DBU. Reagents and conditions are detailed in Table 2.

We have found that in many cases the use of 1,8-diazabicyclo[5.4.0]undec-7-ene (DBU, Scheme 5), rather than CsOH·H_2_O, allows for a more convenient and consistent synthesis with a comparable or higher yield, which is also easier to conduct on a larger scale. DBU is an easily handled liquid which can be directly added to a deprotection reaction in a single portion. In contrast, CsOH·H_2_O is a highly hygroscopic solid which must typically be added dropwise as a solution in (a minimum of) methanol, in which it has low solubility. This dropwise addition is important to minimise deprotection of the tosyl group of MPTTFs, a side reaction which can be caused by the presence of small quantities of methoxide ions in the reaction mixture. As DBU is a non-nucleophilic base, such deprotection cannot occur. Note that unlike in the case of CsOH·H_2_O it is important to heat reactions using DBU to achieve good conversion. Thus, this base is unsuitable if temperature-sensitive moieties are present elsewhere in the molecule. Representative substitutions of MPTTFs [47] with alkyl halides **20**-X [48], **21**-X [49] and **22**-X [50] (where X is a halogen) using both bases are shown in Scheme 5 and Table 2. The products **4i**–**l** are used as building blocks in our work on rotaxanes and psuedorotaxanes [45,50].

**Table 2:** Deprotection and alkylation of cyanoethyl-protected thiol moieties on MPTTFs using CsOH·H_2_O or DBU.

Substrate (R^1^)	RX	Product	Base and conditions	% Yield

**4b** (SEt)	**20**-I	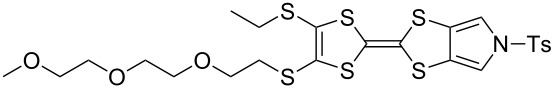 **4i**	CsOH·H_2_O, THF, MeOH, rt, 16 h	92 (lit. 88 [45])
			DBU, THF, reflux, 20 h	90
**4a** (SMe)	**20**-I	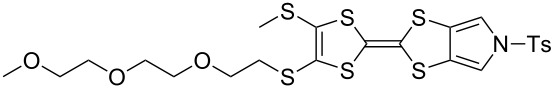 **4j**	CsOH·H_2_O, THF, MeOH, rt, 2.5 h	87
			DBU, THF, reflux, 18 h	91
**4a** (SMe)	**21**-Br	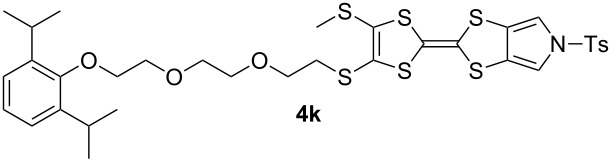	CsOH·H_2_O, THF, MeOH, rt, 20 h	53
**4a** (SMe)	**21**-I		DBU, THF, reflux, 17 h	93
**4a** (SMe)	**22**-I	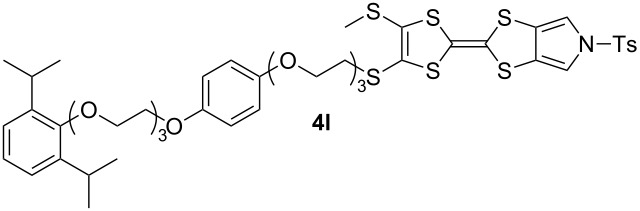	CsOH·H_2_O, THF, MeOH, rt, 18 h	88
			DBU, THF, reflux, 3 d	92

When the preparation of the same product using each of the two bases is compared, it can be seen that the use of DBU rather than CsOH·H_2_O usually results in at least a modest increase in yield. We have prepared **4i** several times with each base and achieved more consistent yields with DBU (typically 88–90%) than with CsOH·H_2_O (typically 80–90%, occasionally lower), although the latter afforded the highest yield of **4i** we have obtained to date (92%). The largest difference in yields is seen for the preparations of **4k**, but it should be noted that the reaction with CsOH·H_2_O used an alkyl bromide whereas that with DBU used the more reactive alkyl iodide analogue. Therefore, this effect may not relate to the change of base alone. In summary, the use of DBU as a deprotecting reagent was found to be high-yielding, consistent and more convenient than the use of CsOH·H_2_O.

#### *N*-Arylation of MPTTFs and BPTTFs

*N*-Alkylation of MPTTFs and BPTTFs can be easily achieved using S_N_2 reactions between a deprotonated pyrrole and a suitably activated aliphatic species [19,28]. *N*-Arylation of MPTTFs and BPTTFs, which allows an annelated TTF to be incorporated into a larger conjugated system, is less common, although interest has increased in recent years. These reactions can be accomplished using a copper-mediated Ullman-type reaction, based on conditions reported by Buchwald and co-workers for the arylation of nitrogen-containing heterocycles and the amidation of aryl halides [51–52]. Examples of TTF derivatives synthesised using this protocol by other groups include: MPTTF and BPTTF-triarylamine conjugates (as possible charge-transport materials) [53], MPTTF-triarylborane conjugates (with possible applications as fluoride sensors) [54], and MPTTF-functionalised calix[4]arenes (which can bind to electron-deficient aromatics and form charge-transfer complexes) [29]. Alternative routes to *N*-arylated MPTTFs proceed through *N*-arylated (1,3)-dithiolo[4,5-*c*]pyrrole-2-ones or (1,3)-dithiolo[4,5-*c*]pyrrole-2-thiones (i.e. analogues of **6**). In some cases these can be prepared similarly to **6** [55], but an alternative route may be required if the desired aryl unit possesses reactive functional groups [12]. These routes are also limited to intermediates that can tolerate the harsh conditions of the subsequent coupling reaction used to form the TTF moiety. Our discussions here will be limited to copper-mediated C–N-bond formation, as we find this to be a flexible and convenient method.

**Scheme 6:**
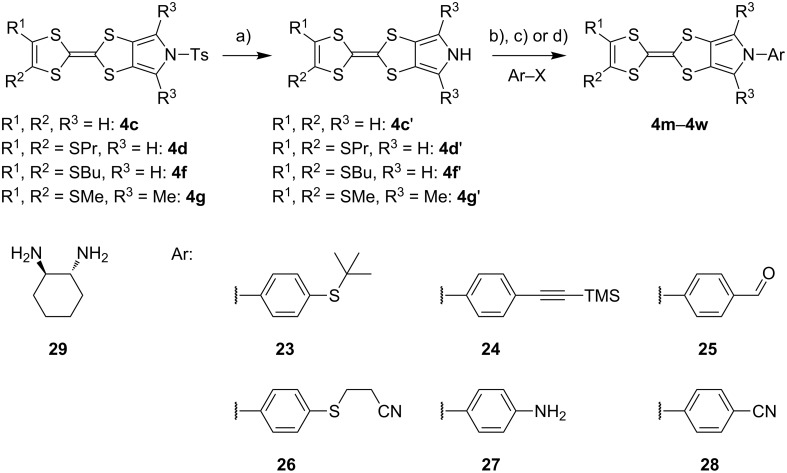
Deprotection and *N*-arylation of tosylated MPTTFs. Reagents and conditions: a) NaOMe, THF, MeOH, reflux, 15 min–6 h, 89–95%; b) CuI, K_3_PO_4_, **29**, THF, 80–115 °C (sealed tube), 3–48 h, 25–68%; c) CuI, K_3_PO_4_, **29**, THF, 100–130 °C (microwave), 2–3 h, 51–93%; d) CuI, K_3_PO_4_, **29**, THF, reflux, 3 h, 67%.

Recent work in our laboratory has involved the *N*-arylation of MPTTFs, including both unsubstituted and thioether-substituted examples (Scheme 6 and Table 3). These materials have served as intermediates and model systems in the synthesis of, for example, donor–acceptor systems, chemosensors and materials with molecular electronics applications [44,56]. The *N*-tosyl protecting group of precursor MPTTFs (**4c**, **4d**, **4f** and **4g**) must first be removed. This can be achieved in excellent yield (89–95%) using sodium methoxide (Scheme 6). The deprotection of MPTTFs derived from **6** proceeded rapidly (15–40 min), whereas that of **4g**, derived from **7**, required 6 h, presumably because of the increased steric bulk of the substituted pyrrole ring. Copper-mediated coupling between the MPTTF products (**4c’**, **4d’**, **4f’** and **4g’**) and a range of aryl halides is then possible. In this study we have investigated several *p*-substituted species (**23**-X–**28**-X, Scheme 6).

**Table 3:** Copper-mediated *N*-arylation of MPTTFs.

MPTTF	Ar-X	Product	Conditions	% Yield

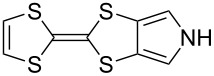 **4c′**	**23**-Br	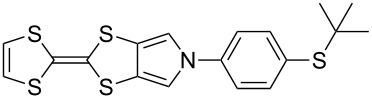 **4m**	ST^a^	35
			MW^b^	81
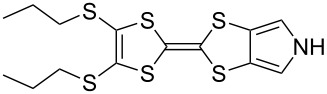 **4d′**	**23**-Br	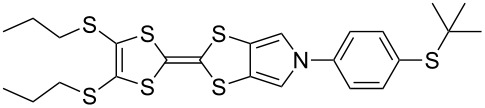 **4n**	ST	68
			MW	93
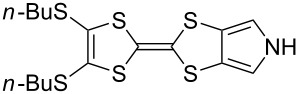 **4f′**	**24**-I	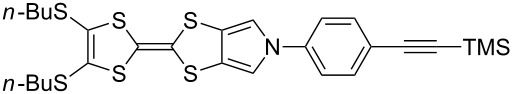 **4o**	Reflux^c^	67
			MW	54
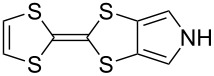 **4c′**	**25**-I	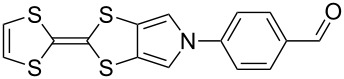 **4p**	ST	35
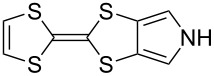 **4c′**	**26**-I	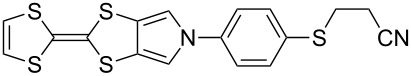 **4q**	ST	52
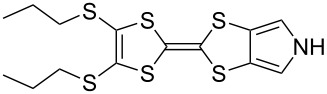 **4d′**	**26**-I	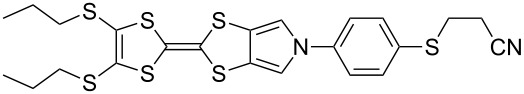 **4r**	ST	65
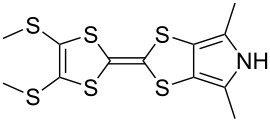 **4g′**	**23**-Br	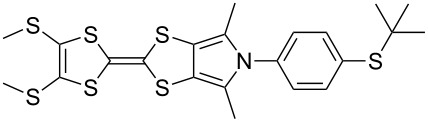 **4s**	ST	25
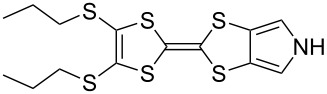 **4d′**	**25**-I	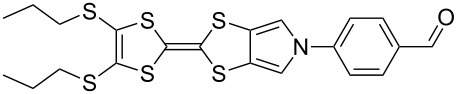 **4t**	MW	82 [56]
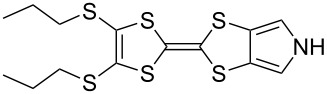 **4d′**	**26**-I	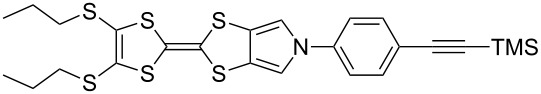 **4u**	MW	51
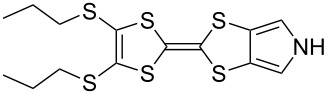 **4d′**	**27**-I	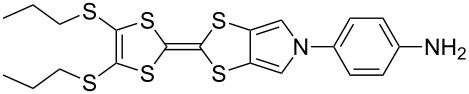 **4v**	MW	80
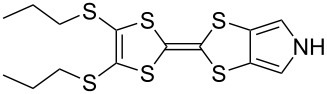 **4d′**	**28**-I	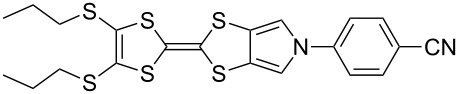 **4w**	MW	64

^a^ST = sealed tube conditions; ^b^MW = microwave conditions; ^c^Reflux = conventional reflux.

As satisfactory results have been reported for similar arylations [29,53–54] when (±)-*trans*-1,2-diaminocyclohexane, **29** (Scheme 6), is used as the ligand, we also followed this approach, rather than using the costlier methylated analogue favoured by the Buchwald group [52]. To improve reaction yields, we made modifications to the published procedures [29,52–54], which typically utilise catalytic CuI in a sealed reaction vessel with 1,4-dioxane as solvent. Firstly, in our hands, comparable yields could be achieved using either THF or 1,4-dioxane as solvent; THF was therefore favoured to facilitate workup. We also saw improvement upon increasing the amount of CuI to 1–2.5 equiv (typically 2 equiv were used). We investigated the use of a microwave reactor (exemplified by the syntheses of **4m** and **4n** in Table 2, which were carried out under both sealed tube and microwave conditions), which typically allowed for shorter reaction times and resulted in higher yields. Small alterations to base and ligand loading and reaction temperature were also made, but with no significant effects. A larger-scale synthesis of **4o** has also been conducted under conventional reflux conditions in good yield.

Scheme 6 and Table 3 show the results of reactions between MPTTFs **4c’**, **4d’**, **4f’** and **4g’** and aryl halides **23**-X–**28**-X to give *N*-arylated products **4m**–**w**. Acceptable yields can be obtained with both aryl bromides and aryl iodides, suggesting that the choice of halogen is not critical. *N*-Arylation of dimethylated MPTTF **4g’** gave the lowest yield amongst the reactions performed under sealed tube conditions, which may be attributable to its more sterically hindered pyrrole-*H*. The highest yielding reactions under sealed tube conditions gave comparable yields to the lowest yielding reactions under microwave conditions. In general, the microwave conditions give high yields and can tolerate a range of functional groups on the aryl halide. We believe that the higher yield of **4o** under conventional reflux as compared to microwave conditions relates to the significant increase in scale. This result indicates that the larger scale synthesis of related species should also be viable.

**Scheme 7:**
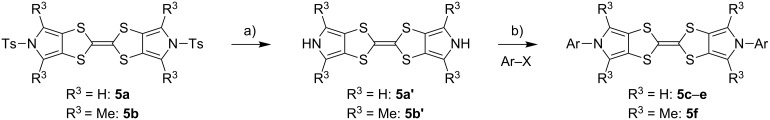
Deprotection and *N*,*N*-diarylation of tosylated BPTTFs. Reagents and conditions: a) NaOMe, THF, MeOH, reflux, 30 min–8 h, 95–99%; b) CuI, K_3_PO_4_, **29**, THF or 1,4-dioxane, 98–110 °C (sealed tube), 22–65 h, 24–72%.

We have begun to extend the sealed tube protocol to the *N*,*N*-diarylation of BPTTFs with promising initial results (Scheme 7 and Table 4). Difficulties were encountered with the isolation, purification and characterisation of the targeted species, which appeared to relate to their extremely poor solubility. Nonetheless, it proved possible to synthesise **5c**–**f** (see Scheme 7 and Table 4), and accomplish some characterisation. The parent deprotected BPTTFs **5a’** and **5b’** were first prepared in near-quantitative yield, comparably to MPTTFs **4c’**, **4d’**, **4f’** and **4g’** (similarly, bulkier **5b’** required a much longer reaction time than **5a’**). In some cases **5a’** was observed to decompose when stored for periods of more than 24 h, although the nature of this decomposition is unclear. Therefore, these materials were prepared and isolated directly before the subsequent coupling reactions, and stored for no longer than 24 h before use. The isolated yields of the *N*,*N*-diarylated BPTTFs compare reasonably with the *N*-arylated MPTTF analogues discussed above, given that two C–N bonds are formed in these syntheses. For these systems we observed larger variation than for MPTTFs when the solvent was changed from 1,4-dioxane to THF; however, a different solvent gave the higher yield in each of the two cases where both solvents were used (**5c** and **5f**). We expect that further optimisation of these reactions can be achieved using microwave or larger scale conditions, and investigations are currently underway in our laboratory.

**Table 4:** *N*,*N*-Diarylation of BPTTFs.

Substrate	Ar–X	Product	% Yield

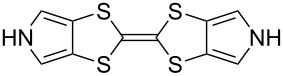 **5a′**	**23**-Br	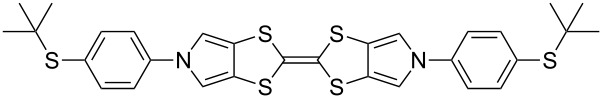 **5c**	42^a^/73^b^
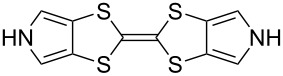 **5a′**	**25**-I	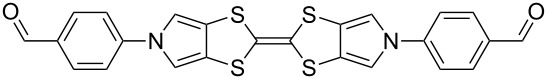 **5d**	45^a^ [56]
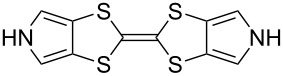 **5a′**	**26**-I	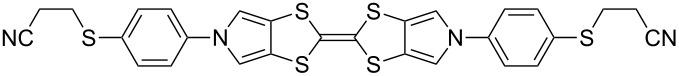 **5e**	24^a^
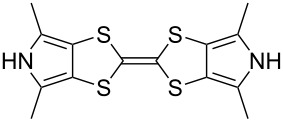 **5b′**	**23**-Br	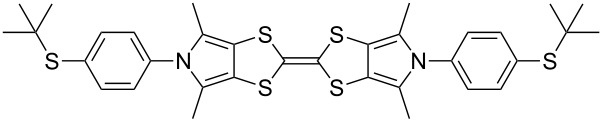 **5f**	40^a^/24^b^

^a^THF as solvent; ^b^1,4-dioxane as solvent.

## Conclusion

Pyrrole-annelated tetrathiafulvalenes (MPTTFs and BPTTFs) are versatile functional groups in many areas of chemistry. The large-scale synthesis of the key intermediate **6** improves the accessibility of these species and their derivatives. The related species **7** can be used to prepare further analogues. Compounds **6** and **7** can both be used to prepare BPTTFs and MPTTFs using homocoupling reactions or cross-coupling reactions with 1,3-dithiole-2-thiones (**19**), respectively. Additional functionalisation of MPTTFs and BPTTFs is important to allow their incorporation into systems with materials applications. The use of the 2-cyanoethyl protecting group allows thioether-functionalised MPTTFs to be prepared, including the addition of large ethylene glycol-based substituents utilised in the preparation of rotaxanes and pseudorotaxanes. *N*-Arylation of MPTTFs is an area of increasing interest and can be achieved using a copper-mediated reaction. We have applied this methodology to a range of aryl halides and achieved improved yields using microwave conditions. Optimisation of this arylation reaction is ongoing in our laboratory, including its extension to BPTTFs. We are continuing to improve and exploit these synthetic routes in our studies of MPTTF and BPTTF-based materials with applications in supramolecular chemistry, molecular electronics and as sensors.

## Supporting Information

File 1 Experimental procedures and analytical data.
