# Supplementary material for: Advances in the synthesis of functionalised pyrrolotetrathiafulvalenes
Source: Beilstein J Org Chem. 2015 Jul 3;11:1112–22. doi: 10.3762/bjoc.11.125 (PMC4505190; doi:10.3762/bjoc.11.125)
Supplement: File 1 — Experimental procedures and analytical data. [file Beilstein_J_Org_Chem-11-1112-s001.pdf]

Supporting Information

for

Advances in the synthesis of functionalised pyrrolotetrathiafulvalenes

Luke J. O'Driscoll, Sissel S. Andersen, Marta V. Solano, Dan Bendixen, Morten Jensen, Troels Duedal, Jess Lycoops, Cornelia van der Pol, Rebecca E. Sørensen, Karina R. Larsen, Kenneth Myntman, Christian Henriksen, Stinne W. Hansen and Jan O. Jeppesen*

Address: Department of Physics, Chemistry and Pharmacy, University of Southern Denmark, Campusvej 55, DK-5230, Odense M, Denmark.

Email: Jan O. Jeppesen - joj@sdu.dk

* Corresponding author

Experimental procedures and analytical data

General

All reactions were carried out under an atmosphere of anhydrous N_2, unless otherwise stated. MeOH was distilled from Mg, while THF was distilled from molecular sieves (4 Å) immediately prior to use. $(EtO)_3P$ was distilled and stored over molecular sieves (4 Å). Other anhydrous solvents were allowed to stand over molecular sieves (4 Å) for at least three days prior to use. All used reagents were standard grade and used as received, except sodium tosylamide (**13**) [1], 2,5-dimethyl-3,4-dithiocyanopyrrole (**16**) [2,3], 1,3-dithiole-2-thiones (**19**) (see for example Simonsen et al. [4]), **20**-I [5], **21**-X (X = Br and X = I can be prepared comparably to the analogous tosylate [6]), **22**-I [7], **23**-Br [8] and **26**-I [9], which were all prepared based on literature procedures. Thin layer chromatography (TLC) was carried out using aluminium sheets pre-coated with silica gel 60F (Merck F_{254}) and visualisation was achieved using UV light (254 nm) or I_2. Column chromatography was conducted using silica gel (ROCC 40–60 µm). Melting points (mp) were determined on a Büchi melting point apparatus and are uncorrected. ^{1}H and ^{13}C NMR spectra were recorded either at room temperature on a Bruker AVANCE III spectrometer (400 MHz and 100 MHz, respectively), at 298 K on a Bruker instrument with a non-inverse cryoprobe (500 MHz and 125 MHz, respectively), or at 298 K on a Varian Gemini spectrometer (300 MHz and 75 MHz, respectively). Chemical shifts are quoted on the δ scale and coupling constants (*J*) are expressed in Hertz (Hz). Samples were prepared using solvents purchased from Sigma–Aldrich and all spectra were referenced using the residual solvent peak as an internal standard. Electrospray ionization (ESI) mass spectra were recorded on a Bruker Daltonics micrOTOF-Q II ESI-Qq_TOF mass spectrometer. Matrix-assisted laser desorption

ionisation (MALDI) mass spectra were recorded on a Bruker Autoflex III Smartbeam instrument. Electron impact (EI) mass spectra were recorded on a Finnegan MAT TSQ 700 Triple Stage Quadrupole instrument. IR spectra were recorded on a Perkin Elmer 1720 infrared Fourier transform spectrophotometer. Elemental analyses were performed by Atlantic Microlabs, Inc., Atlanta, Georgia or at the University of Copenhagen.

In the experimental procedures section, petroleum ether refers to petroleum ether bp 60–80 °C. Column chromatography eluent mixtures are given as v/v ratios. Where deactivated silica was used as a stationary phase, it was prepared based on the following procedure. SiO_2 (1 L) was suspended in CH_2Cl_2. Et_3N (30 mL) was added and the suspension stirred for 1 h. The silica was isolated by filtration, washed with CH_2Cl_2 and allowed to dry overnight before use or storage in a sealed container. The synthesis of the following materials is reported elsewhere: **17** [10], **7** [10], **5b** [10], **4f** [11], **4g** [10], **4h** [12], **4f'** [11], **4g'** [10], **4t** [13], **5b'** [10] and **5d** [13].

Experimental procedures

4,5-Bis(methoxycarbonyl)-1,3-dithiole-2-thione (8):

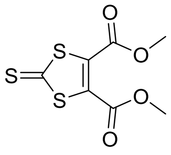

8

Ethylene trithiocarbonate (**9**) (101 g, 743 mmol) and dimethyl acetylenedicarboxylate (**10**) (178 g, 710 mmol) were dissolved in toluene (260 mL). The yellow solution was refluxed for 19 h (135 °C) causing a color change to orange/brown. The solution was then cooled to room temperature using an ice water bath and petroleum ether (160 mL) was added slowly to precipitate the product, which was collected by

filtration and washed with petroleum ether (2 × 50 mL) before drying in vacuo to afford yellow crystals of **8** (132 g, 529 mmol, 74%). Mp 83–84 °C (lit. [14] 86–87 °C); ^{1}H NMR (300 MHz, $CDCl_3$): δ 3.91 (s, 6H); ^{13}C NMR (75 MHz, $CDCl_3$): δ 207.3, 158.0, 138.1, 53.8; MS-EI$^+$ m/z: 252 ([M + 2]$^+$, 12), 251 ([M + 1]$^+$, 17), 250 (M^+, 100), 219 (15), 191 (8), 174 (13), 107 (18), 87 (14), 84 (18), 59 (36); IR (KBr) ν/cm^{-1}: 1746, 1723, 1258, 1087.

4,5-Bis(hydroxymethyl)-1,3-dithiole-2-thione (11):

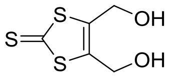

11

Compound **8** (40.0 g, 160 mmol) and oven-dried LiBr (28.0 g, 320 mmol) were added to a mixture of anhydrous THF (400 mL) and anhydrous MeOH (150 mL) in a dry flask fitted with a mechanical stirrer and cooled to −10 °C under stirring. Powdered $NaBH_4$ (24.2 g, 640 mmol) was added in portions over 2 h, ensuring the reaction temperature remained below −5 °C. The stirred reaction mixture was warmed to −5 °C over the next 4 h and then allowed to slowly reach room temperature overnight. Water (600 mL) and ice (400 mL) were added to the reaction mixture, followed by the addition of 4 M aqueous HCl solution until the mixture was acidic. The mixture was extracted with EtOAc (160 mL, then 7 × 80 mL) and the combined organic phases were washed with H_2O (2 × 250 mL) and dried ($MgSO_4$). After filtration, evaporation of the solvent gave an orange oil, which was dissolved in a minimum of THF (~150 mL) to which celite (100 mL) was added. After removing the solvent the product was purified by column chromatography (SiO_2, CH_2Cl_2/EtOAc 2:1–1:1 (following elution of the first yellow band) – EtOAc (after collecting ~8.5 L of product-containing fractions)), by depositing the crude product suspended on celite

onto the top of the silica. Removal of solvent afforded yellow crystals of **11** (23.9 g, 123 mmol, 77%). ^{1}H NMR (400 MHz, $(CD_3)_2SO$): δ 5.87 (t, J = 5.5 Hz, 2H), 4.45 (d, J = 5.5 Hz, 4H); ^{13}C NMR (100 MHz, $(CD_3)_2SO$): δ 212.3, 142.6, 56.7.

4,5-Bis(bromomethyl)-1,3-dithiole-2-thione (12):

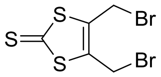

12

The dialcohol **11** (36.1 g, 186 mmol) was dissolved in anhydrous THF (400 mL). The solution was cooled to 0 °C and PBr_3 (41 mL, 118.1 g, 436 mmol) was added over 1 h. The reaction was cooled for another 30 min and the reaction mixture was then stirred for 20 h at room temperature. The reaction mixture was evaporated to give an orange-brown oil to which cold MeOH (0 °C, 350 mL) was added, causing precipitation of yellow crystals. The mixture was left in the freezer for 6 h, before compound **12** was isolated by filtration (44.7 g, 140 mmol, 75%), mp 126.5 – 128.5 ^{o}C (lit. [12] 126 – 126.5 ^{o}C). ^{1}H NMR (400 MHz, $CDCl_3$): δ 4.33 (s, 4H); ^{13}C NMR (100 MHz, $CDCl_3$): δ 208.0, 139.4, 20.3; MS-EI^{+} m/z: 322 ([M + 4]$^{+}$, 10), 320 ([M + 2]$^{+}$, 40), 318 (M^{+}, 21), 241 ([M + 2]$^{+}$ – Br, 88), 239 (M^{+} – Br, 74), 165 (26), 163 (30), 160 (10), 84 (100), 76 (42), 58 (78), 50 (37), 45 (34), 39 (40), 32 (10); IR (KBr) v/cm^{-1}: 1201, 1064, 595.

4,6-Dihydro-*N*-tosyl-(1,3)-dithiolo[4,5-*c*]pyrrole-2-thione (14) (intermediate):

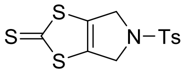

14

Sodium tosylamide (**13**) (55.9 g, 289 mmol) was suspended in anhydrous MeCN (1 L) and heated to 80 °C. Dibromide **12** (44.3 g, 138 mmol) was dissolved in

anhydrous DMF (200 mL) and added to the suspension over 15 min. After stirring the reaction mixture for a further 15 min at 80 °C, it was slowly poured into a stirred mixture of ice–H_2O (2.5 L). The light brown suspension was stirred for 60 min before isolating the crude product by filtration. The obtained solid was washed with MeOH (1.4 L) and dried to afford crude **14** (36.8 g, 112 mmol), which was used without further purification.

4,6-Dihydro-*N*-tosyl-(1,3)-dithiolo[4,5-*c*]pyrrole-2-one (15) (intermediate):

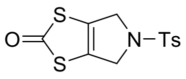

15

Crude **14** (36.8 g, 112 mmol) was suspended in $CHCl_3$[1] (1 L) and glacial acetic acid (100 mL). $Hg(OAc)_2$ (62.2 g, 195 mmol) was added and the reaction mixture was stirred at room temperature for 24 h. Afterwards the mixture was filtered through Celite 545 (500 mL) and washed with CH_2Cl_2 (8 × 100 mL). The filtrate was washed with a saturated aqueous solution of $NaHCO_3$ (6 × 250 mL), then H_2O (2 × 250 mL) and dried ($MgSO_4$). Removal of the solvent gave **15** as a dark solid (29.0 g, 93 mmol) which was used without further purification.

***N*-Tosyl-(1,3)-dithiolo[4,5-*c*]pyrrole-2-one (6):**

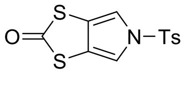

6

Crude **15** (29.0 g, 93 mmol) and DDQ (33.2 g, 146 mmol) were dissolved in PhCl (800 mL) and the mixture was heated to reflux for 4 h then cooled to room

[1] We recommend that this reaction be conducted in CH_2Cl_2, however, it was unavailable to us when this synthesis was performed. Therefore chloroform was used instead with no significant effect from the solvent change.

temperature. The solvent was removed and the solid residue was dissolved in CH_2Cl_2 (500 mL) to which SiO_2 (150 mL) was added before removing the solvent. The compound was purified by column chromatography (SiO_2, CH_2Cl_2/petroleum ether 1:1) by depositing the crude product suspended on silica onto the top of the column. Compound **6** was isolated as a white solid (22.5 g, 72.3 mmol, 52% from **12**), mp 180.5 – 182 °C (lit. [15] 178.5 – 179 °C). ^{1}H NMR (400 MHz, $CDCl_3$): δ 8.34 (d, J = 8.5 Hz, 2H), 7.78 (d, J = 8.5 Hz, 2H), 7.22 (s, 2H), 2.43 (s, 3H); ^{13}C NMR (100 MHz, $CDCl_3$): δ 193.7, 146.0, 135.1, 130.3, 127.2, 119.5, 112.8, 21.7; MS-EI$^+$ *m*/*z*: 311 (M^+, 59), 283 (31), 155 (Ts^+, 67), 91 (tropylium ion, 100); IR (KBr) v/cm^{-1}: 3130, 1716, 1646, 1596, 1370, 1236, 1172, 1091, 1067.

Bis(*N*-tosylpyrrolo-[3,4-*d*])tetrathiafulvalene (5a):

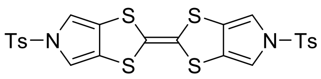

5a

Compound **6** (2.00 g, 6.42 mmol) was dissolved in freshly distilled $(EtO)_3P$ (70 mL) and degassed for 15 min under argon atmosphere. The mixture was heated to 130 °C for 5.5 h before the reaction mixture was allowed to cool to room temperature. Addition of cold MeOH (120 mL) afforded a yellow precipitate, which was removed by filtration, washed with MeOH (2 × 20 mL) and dried in vacuo to afford **5a** as a yellow solid with no further purification (1.46 g, 2.47 mmol, 76%), mp > 250 °C (lit. [12] > 250 °C). ^{1}H NMR (400 MHz, $(CD_3)_2SO$): δ 7.81 (d, J = 8.2 Hz, 4H), 7.45 (d, J = 8.2 Hz, 4H), 7.38 (s, 4H), 2.38 (6H, s); MS-MALDI *m/z*: 590 (M^+, 48), 435 ($[M - Ts]^+$, 18) 280 ($[M – 2 \times Ts]^+$, 12).

***N*-Tosyl-2-[4-(methylthio)-5-(2-cyanoethylthio)-(1,3)-dithiole-2-ylidene]-(1,3)-dithiolo[4,5-*c*]pyrrole (4a):**

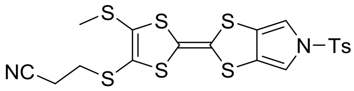

4a

Compounds **6** (1.80 g, 5.78 mmol) and **19a** (2.28 g, 8.58 mmol) were dissolved in $(EtO)_3P$ (50 mL) and heated to 135 °C. After 12 min, additional **19a** (0.74 g, 2.79 mmol) was added to the reaction mixture and after another 6 min, another portion of **19a** (0.73 g, 2.75 mmol) was added. After heating at 135 °C for 2.5 h, the reaction mixture was cooled to room temperature. MeOH (75 mL) was added and the mixture was stored in a freezer for 45 min, affording a precipitate, which was isolated by filtration and dried. The filtrate was concentrated to 1/3 of its volume and additional MeOH (50 mL) was added and the mixture was stored in the freezer overnight, affording additional precipitate which was also isolated by filtration and dried. This procedure was repeated twice. The combined solids were dissolved in CH_2Cl_2 (250 mL) to which SiO_2 (60 mL) was added before removing the solvent. The compound was purified by column chromatography (SiO_2, CH_2Cl_2) by depositing the crude product suspended on silica onto the top of the column. The isolated product was dried before crystallising by dissolving in CH_2Cl_2 (500 mL) and adding MeOH (300 mL). The resulting suspension was concentrated to half of this volume and the precipitate isolated by filtration. The yellow solid was washed with cold MeOH (0 °C, 4 × 25 mL) and dried to afford **4a** as a yellow solid (2.60 g, 4.93 mmol, 85%), mp 194 – 194.5 °C. ^{1}H NMR (400 MHz, $CDCl_3$): δ 7.73 (d, J = 8.4 Hz, 2H), 7.30 (d, J = 8.4 Hz, 2H), 6.95 (s, 2H), 3.01 (t, J = 7.2 Hz, 2H), 2.68 (t, J = 7.2 Hz, 2H), 2.46 (s, 3H), 2.42 (s, 3H); ^{13}C NMR (100 MHz, $CDCl_3$): δ 145.7, 135.5, 135.0, 130.3, 127.1, 127.0,

119.9, 117.6, 113.5, 111.5, 31.4, 21.8, 19.2, 18.8; MS-ESI$^+$ *m/z*: 529 (M^+); 528 ([M – 1]$^+$)

N-Tosyl-2-[4-(ethylthio)-5-(2-cyanoethylthio)-(1,3)-dithiole-2-ylidene]-(1,3)-dithiolo[4,5-*c*]pyrrole (4b):

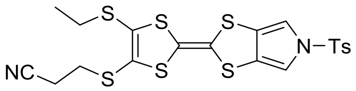

4b

Compounds **6** (2.00 g, 6.42 mmol) and **19b** (2.79 g, 9.98 mmol) were dissolved in $(EtO)_3P$ (45 mL) which was preheated to 130 °C. After 7 min, an additional portion of **19b** (0.95 g, 3.40 mmol) was added and after another 7 min, another portion of **19b** (0.74 g, 2.6 mmol) was added. After heating for 90 min at 130 °C, the reaction mixture was cooled to room temperature. Cold MeOH (0 °C, 100 mL) was added, affording a precipitate which was isolated by filtration and washed with cold MeOH (0 °C, 50 mL). The precipitate was dissolved in CH_2Cl_2 (300 mL) to which SiO_2 (50 mL) was added before removing the solvent. The compound was purified by column chromatography (SiO_2, CH_2Cl_2/cyclohexane 4:1) by depositing the crude product suspended on silica onto the top of the column. Compound **4b** was obtained as a yellow solid (2.89 g, 5.32 mmol, 83%), mp 200 – 201 °C (lit. [16] 200 – 201 °C). ^{1}H NMR (400 MHz, $(CD_3)_2SO$): δ 7.77–7.87 (m, 2H), 7.42–7.51 (m, 2H), 7.39 (s, 2H), 3.11 (t, J = 6.6 Hz, 2H), 2.89 (q, J = 7.3 Hz, 2H), 2.81 (t, J = 6.6 Hz, 2H), 2.38 (s, 3H), 1.23 (t, J = 7.3 Hz, 3H); ^{13}C NMR (100 MHz, $(CD_3)_2SO$): δ 145.8, 134.4, 130.4, 129.8, 126.8, 126.0, 125.9, 124.0, 118.7, 117.7, 112.7, 112.3, 30.9, 29.9, 21.1, 18.1, 14.9; MS-MALDI *m*/*z*: 542.06 (M^+).

***N*-Tosyl-2-((1,3)-dithiole-2-ylidene)-(1,3)-dithiolo[4,5-*c*]-pyrrole (4c):**

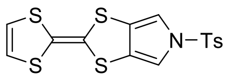

4c

A degassed solution of **19c** (0.49 g, 3.65 mmol) and **6** (0.75 g, 2.43 mmol) dissolved in freshly distilled $(EtO)_3P$ (30 mL) was heated to 130 °C. After 10 min an additional portion of **19c** (0.49 g, 3.65 mmol) was added. After heating for 3 h at 130 °C, the reaction mixture was cooled to room temperature. Cold MeOH (0 °C, 50 mL) was added, affording a yellow precipitate, which was isolated by filtration and washed with MeOH (3 × 15 mL). The solid was purified by column chromatography (SiO_2, CH_2Cl_2/petroleum ether 1:2 – 1:1) to afford **4c** (0.68 g, 1.72 mmol, 71%) as a yellow solid, mp > 250°C (lit. [17] > 250 ^{o}C). ^{1}H NMR (400 MHz, $(CD_3)_2SO$): δ 7.83 (d, J = 8.2 Hz, 2H), 7.46 (d, J = 8.2 Hz, 2H), 7.36 (s, 2H), 6.74 (s, 2H), 2.38 (s, 3H); ^{13}C NMR (100 MHz, $(CD_3)_2SO$): δ 145.8, 134.5, 130.4, 126.9, 126.7, 119.6, 118.7, 112.7, 112.4, 21.1; MS-EI^{+} m/z: 394.96 (M^{+}, 100); IR (KBr) v/cm^{-1}: 3137, 1635, 1596, 1370, 1225, 1172, 1090, 1055, 680; Anal. Calcd for $C_{15}H_{11}NO_2S_5$: C, 45.31; H, 2.79; N, 3.52. Found: C, 44.82; H, 2.86; N, 3.40.

***N*-Tosyl-2-(4,5-bis(propylthio)-(1,3)-dithiole-2-ylidene)-(1,3)-dithiolo[4,5-*c*]pyrrole (4d):**

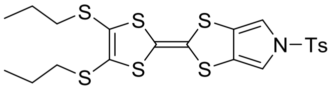

4d

Compounds **19d** (0.89 g, 3.15 mmol) and **6** (0.65 g, 2.09 mmol) were dissolved in $(EtO)_3P$ and heated to 130 °C. After 7 min, additional **19d** (0.29 g, 1.04 mmol) was added to the reaction mixture and after another 7 min, another portion of **19d** (0.29 g, 1.04 mmol) was added. After heating for 1 h at 130 °C, the reaction mixture

was cooled to room temperature. Cold MeOH (0 °C, 50 mL) was added and the mixture was then stored in the freezer overnight, affording a yellow precipitate which was isolated by filtration and washed with cold MeOH (0 °C, 2 × 50 mL). The precipitate was dissolved in CH_2Cl_2 (50 mL) to which SiO_2 (20 mL) was added before removing the solvent. The compound was purified by column chromatography (SiO_2, CH_2Cl_2:petroleum ether 1:1) by depositing the crude product suspended on silica onto the top of the column. Compound **4d** was obtained as a yellow solid (0.99 g, 1.81 mmol, 87%), mp 115.5 – 116.5 °C (lit. [18] 132.0 – 132.5 °C). ^{1}H NMR (400 MHz, $CDCl_3$): δ 7.72 (d, J = 8.2 Hz, 2H), 7.29 (d, J = 8.2 Hz, 2H), 6.93 (s, 2H), 2.78 (t, J = 7.3 Hz, 4H), 2.41 (s, 3H), 1.64 (sextet, J = 7.3 Hz, 4H), 1.00 (t, J = 7.3 Hz, 6H); ^{13}C NMR (100 MHz, $CDCl_3$): δ 145.5, 135.4, 130.1, 127.5, 127.3, 127.0, 116.3, 115.3, 111.3, 38.3, 23.1, 21.7, 13.2; MS-ESI$^+$ *m/z:* 545 (M^+), 391 ($[M – Ts]^+$).

***N*-Tosyl-2-(4,5-bis(2-cyanoethylthio)-(1,3)-dithiole-2-ylidene)-(1,3)-dithiolo[4,5-*c*]pyrrole (4e):**

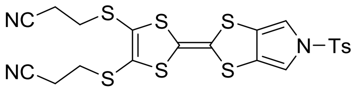

4e

Compounds **6** (1.68 g, 5.38 mmol) and **19e** [4] (2.67 g, 8.76 mmol) were dissolved in $(EtO)_3P$ (50 mL) which was preheated to 130 °C. After 10 min, additional **19e** (1.14, 3.76 mmol) was added and after another 10 min, another portion of **19e** (0.82 g, 2.69 mmol) was added. After heating for 90 min at 130 °C, the reaction mixture was cooled to room temperature. Cold MeOH (0 °C, 150 mL) was added and the mixture was then stored in the freezer for 1 h, affording an orange precipitate which was isolated by filtration and washed with MeOH (3 × 25 mL). The precipitate was purified by column chromatography (SiO_2, CH_2Cl_2/cyclohexane 4:1). Compound

4e was obtained as a yellow solid (2.15 g, 3.78 mmol, 70%), mp 180.5 – 181.5 °C (lit. [12] 177 – 178 °C). ^{1}H NMR (400 MHz, $(CD_3)_2SO$): *δ* 7.82 (d, *J* = 8.4 Hz, 2H), 7.45 (d, *J* = 8.1 Hz, 2H), 7.39 (s, 2H), 3.14 (t, *J* = 6.6 Hz, 4H), 2.86 (t, *J* = 6.8 Hz, 4H), 2.38 (s, 3H).

***N*-Tosyl-2-[4-(ethylthio)-5-(2-(2-(2-methoxyethoxy)ethoxy)ethylthio)-(1,3)-dithiole-2-ylidene]-(1,3)-dithiolo[4,5-*c*]pyrrole (4i):**

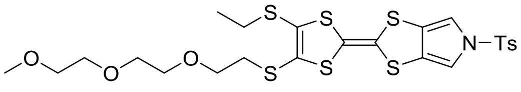

4i

Method A:

Compound **4b** (402 mg, 0.74 mmol) was dissolved in anhydrous THF (150 mL) and degassed (N_2, 20 min). $CsOH•H_2O$ (132 mg, 0.79 mmol) dissolved in anhydrous MeOH (0.5 mL) was added dropwise over 1 h to the stirred solution. After a further 1 h **20**-I (223 mg, 0.81 mmol) was added and the reaction mixture was stirred for 16 h at room temperature. The solvent was then removed in vacuo giving a brown oil which was redissolved in CH_2Cl_2 (100 mL), washed with H_2O (3 × 200 mL) and dried ($MgSO_4$). After filtration and removal of the solvent the compound was purified by column chromatography (SiO_2, CH_2Cl_2:EtOAc 10:1), to afford **4i** as a brown oil (433 mg, 0.68 mmol, 92%). ^{1}H NMR (400 MHz, $CDCl_3$): *δ* 7.72 (d, *J* = 8.3 Hz, 2H), 7.29 (d, *J* = 8.3 Hz, 2H), 6.93 (s, 2H), 3.62–3.67 (m, 8H), 3.52–3.54 (m, 2H), 3.36 (s, 3H), 2.99 (t, *J* = 6.8 Hz, 2H), 2.83 (q, *J* = 7.4 Hz, 2H), 2.41 (s, 3H), 1.29 (t, *J* = 7.4 Hz, 3H); MS-ESI$^+$ *m*/*z*: 635 (M^+); 636 ($[M + H]^+$), 657 ($[M + Na]^+$); IR (NaCl) *v*/cm^{-1}: 3137, 2870, 1625, 1374, 1227, 1188, 1174, 1091, 1054.

Method B:

Compounds **4b** (997 mg, 1.84 mmol) and **20**-I (500 mg, 1.82 mmol) were dissolved in anhydrous THF (250 mL) and degassed (N_2, 20 min), before DBU (0.55 mL, 560 mg, 3.68 mmol) was added and the reaction mixture was heated to reflux. After 20 h the reaction mixture was cooled to room temperature and the solvent was removed in vacuo, affording a brown oil which was redissolved in CH_2Cl_2 (300 mL), washed with H_2O (3 × 200 mL) and dried ($MgSO_4$). After filtration and removal of the solvent the compound was purified by column chromatography (SiO_2, CH_2Cl_2/EtOAc 10:1) to afford **4i** as a brown oil (1040 mg, 1.64 mmol, 90%). Characterisation was in agreement with the data given for Method A above.

***N*-Tosyl-2-[4-(methylthio)-5-(2-(2-(2-methoxyethoxy)ethoxy)ethylthio)-(1,3)-dithiole-2-ylidene]-(1,3)-dithiolo[4,5-*c*]pyrrole (4j):**

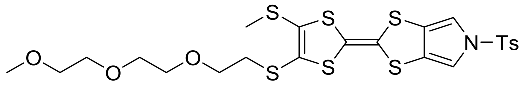

4j

Method A:

Compound **4a** (515 mg, 0.97 mmol) was dissolved in anhydrous THF (70 mL) and degassed (N_2, 20 min). $CsOH•H_2O$ (166 mg, 0.99 mmol) dissolved in anhydrous MeOH (1.2 mL) was added dropwise over 1 h to the stirred solution. Afterwards **20**-I (284 mg, 1.04 mmol) was added and the reaction mixture was stirred for an additional 2.5 h at room temperature. The solvent was then removed in vacuo giving a brown oil which was redissolved in CH_2Cl_2 (200 mL), washed with H_2O (2 × 150 mL) and dried ($MgSO_4$). After filtration and removal of the solvent the compound was purified by column chromatography (SiO_2, CH_2Cl_2:EtOAc 2:1), to afford **4j** as a yellow/brown oil (524 mg, 0.84 mmol, 87%).^{1}H NMR (400 MHz, $CDCl_3$) δ 7.72 (d, J =

8.0 Hz, 2H), 7.30 (d, *J* = 8.0 Hz, 2H), 6.93 (s, 2H), 3.66 (t, *J* = 6.8 Hz, 2H), 3.62–3.65 (m, 6H), 3.52–3.55 (m, 2H), 3.36 (s, 3H), 2.98 (t, *J* = 6.8 Hz, 2H), 2.41 (s, 6H); MS-ESI$^+$ *m*/*z*: 644 ([*M* + Na]$^+$), 621 (M^+); HRMS (FT-ESI) calcd. for $C_{23}H_{27}NO_5S_7^+$: 620.9934. Found: 620.9955; Anal. Calcd for $C_{23}H_{27}NO_5S_7$: C, 44.42; H, 4.36; N, 2.25; S, 36.09. Found: C, 44.65; H, 4.37; N, 2.23; S, 36.32.

Method B:

Compounds **4a** (332 mg, 0.63 mmol) and **20**-I (162 mg, 0.60 mmol) were dissolved in anhydrous THF (60 mL) and degassed (N_2, 20 min), before DBU (0.19 mL, 193 mg, 1.27 mmol) was added and the reaction mixture was heated to reflux. After 18 h the reaction mixture was cooled to room temperature and the solvent was removed in vacuo, affording a yellow oil which was redissolved in CH_2Cl_2 (70 mL), washed with H_2O (3 × 100 mL) and dried ($MgSO_4$). After filtration and removal of the solvent the compound was purified by column chromatography (SiO_2, CH_2Cl_2 – CH_2Cl_2/EtOAc 10:1), to afford **4j** as a yellow oil (340 mg, 0.55 mmol, 91%). Characterisation was in agreement with the data given for Method A above.

***N*-Tosyl-2-[4-(methylthio)-5-(2-(2-(2-(2,6-diisopropylphenyloxy)ethoxy)ethoxy)ethylthio)-(1,3)-dithiole-2-ylidene]-(1,3)-dithiolo[4,5-*c*]pyrrole (4k):**

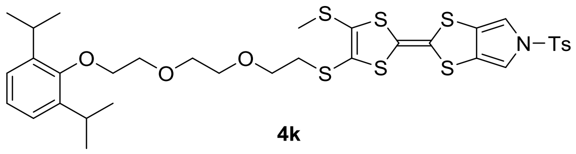

4k

Method A:

Compound **4a** (265 mg, 0.50 mmol) was dissolved in anhydrous THF (50 mL) and degassed (N_2, 20 min) followed by the addition of **21**-Br (189 mg, 0.51 mmol). Afterwards a solution of $CsOH{\bullet}H_2O$ (92 mg, 0.55 mmol) in anhydrous MeOH (0.5 mL) was added dropwise over 60 min to the stirred solution. The reaction

mixture was stirred for a further 20 h at room temperature. The solvent was removed in vacuo, giving a brown oil, which was redissolved in CH_2Cl_2 (100 mL), washed with H_2O (3 × 100 mL), and dried ($MgSO_4$). After filtration and removal of the solvent the compound was purified by repeated column chromatography (SiO_2, CH_2Cl_2/cyclohexane 2:1 – CH_2Cl_2), to afford **4k** as a yellow oil (204 mg, 0.27 mmol, 53%). Characterisation was in agreement with the data given for Method B below.

Method B:

Compounds **4a** (170 mg, 0.32 mmol) and **21**-I (127 mg, 0.30 mmol) were dissolved in anhydrous THF (30 mL) and degassed (N_2, 15 min), before DBU (0.095 mL, 97 mg, 0.64 mmol) was added and the reaction mixture was heated to reflux. After 17 h the reaction mixture was cooled to room temperature and the solvent was removed in vacuo, affording a yellow oil which was redissolved in CH_2Cl_2 (100 mL), washed with H_2O (3 × 100 mL), and dried ($MgSO_4$). After filtration and removal of the solvent the compound was purified by column chromatography (SiO_2, CH_2Cl_2 – CH_2Cl_2/EtOAc 10:1), to afford **4k** as a yellow oil (214 mg, 0.28 mmol, 93%). ^{1}H NMR (400 MHz, $CDCl_3$): δ 7.72 (d, J = 8.4 Hz, 2H), 7.29 (d, J = 8.4 Hz, 2H), 7.09 (s, 3H), 6.92 (q, J = 2.0 Hz, 2H), 3.90-3.92 (m, 2H), 3.84-3.86 (m, 2H), 3.69-3.76 (m, 6H), 3.37 (septet, J = 6.9 Hz, 2H), 3.00 (t, 2H, J = 6.7 Hz), 2.41 (s, 6H), 1.21 (d, J = 6.9 Hz, 12H); ^{13}C NMR (100 MHz, $CDCl_3$): δ 153.1, 145.5, 141.8, 135.4, 130.2, 127.2, 127.1, 127.0, 124.6, 124.0, 111.3, 73.9, 70.9, 70.71, 70.66, 70.1, 35.5, 26.3, 24.2, 21.7, 19.1; MS-MALDI *m/z*: 790 ([M + Na]$^+$, 29); 770 ([M + 3]$^+$, 12); 769 ([M + 2]$^+$, 42); 768 ([M + 1]$^+$, 48); 767([M +]$^+$, 100); HRMS (FT-ESI) calcd. for $C_{34}H_{41}NO_5S_7Na^+$: 790.0922. Found: 790.0942; Anal. Calcd for $C_{34}H_{41}NO_5S_7$: C, 53.16; H, 5.38; N, 1.82; S, 29.22. Found: C, 53.39; H, 5.43; N, 1.88; S, 28.96.

N-Tosyl-2-[4-(methylthio)-5-(2-(2-(2-(4-(2-(2-(2-(2,6-diisopropylphenyloxy)ethoxy)ethoxy)ethoxy)phenyloxy)ethoxy)ethoxy)ethylthio)-(1,3)-dithiole-2-ylidene]-(1,3)-dithiolo[4,5-c]pyrrole (4l):

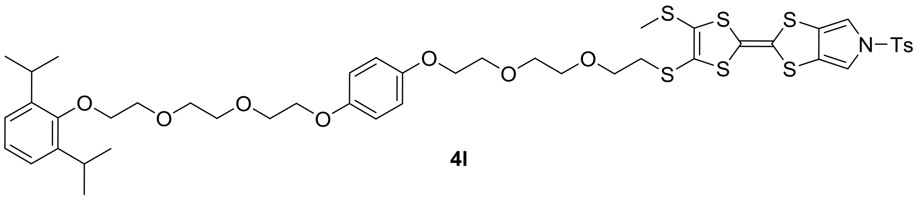

4l

Method A:

Compounds **4a** (491 mg, 0.93 mmol) and **22**-I (619 mg, 0.96 mmol) were dissolved in anhydrous THF and degassed (N_2, 15 min). A solution of $CsOH•H_2O$ (162 mg, 0.96 mmol) in anhydrous MeOH (0.5 mL) and anhydrous THF (1.5 mL) was added dropwise over 4 h to the stirred solution. The reaction mixture was stirred for a further 14 h at room temperature. The solvent was removed in vacuo, giving an oil which was redissolved in CH_2Cl_2 (100 mL), washed with H_2O (3 × 150 mL) and dried ($MgSO_4$). After filtration and removal of the solvent the compound was purified by column chromatography (SiO_2, CH_2Cl_2 – CH_2Cl_2/EtOAc 1:1), to afford **4l** as a yellow oil (813 mg, 0.82 mmol, 88%). ^{1}H NMR (400 MHz, $CDCl_3$): δ 7.69–7.74 (m, 2H), 7.27–7.31 (m, 2H), 7.09 (s, 3H), 6.92 (AB, J = 2.2 Hz, 2H), 6.83 (s, 4H), 4.04–4.08 (m, 4H), 3.64–3,93 (m, 18H), 3.39 (septet, J = 6.9 Hz, 2H), 2.96–3.00 (m, 2H), 2.41 (s, 3H), 2.40 (s, 3H), 1.21 (d, J = 6.9 Hz, 12H); MS-ESI$^+$ m/z: 992 (M^+); HRMS (FT ESI) calcd. for $C_{46}H_{57}NO_9S_7^+$ 991.2078. Found: 992.2151; Anal. Calcd for $C_{46}H_{57}NO_9S_7$: C, 55.67; H, 5.79; N, 1.41. Found: C, 55.98; H, 5.85; N, 1.24.

Method B:

MPTTF **4a** (336 mg, 0.64 mmol) and glycol chain **22**-I (398 mg, 0.62 mmol) were dissolved in anhydrous THF (100 mL) and degassed (N_2, 20 min), before DBU (193 mg, 0.19 mL, 1.27 mmol) was added and the reaction mixture was heated to

reflux. After 3 days the reaction was cooled to room temperature and the solvent was removed in vacuo, affording an oil which was redissolved in CH_2Cl_2 (100 mL), washed with H_2O (3 × 150 mL) and dried ($MgSO_4$). After filtration and removal of the solvent the compound was purified by column chromatography (SiO_2, CH_2Cl_2/EtOAc 1:1), to afford **4l** as a yellow oil (570 mg, 0.54 mmol, 92%). Characterisation was in agreement with the data given for Method A above.

2-((1,3)-Dithiole-2-ylidene)-(1,3)-dithiolo[4,5-*c*]-pyrrole (4c'):

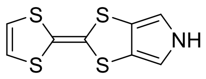

4c'

Compound **4c** (0.46 g, 1.15 mmol) was dissolved in a mixture of anhydrous THF (12 mL) and anhydrous MeOH (12 mL). The solution was degassed (Ar, 20 min) then placed under an argon atmosphere before NaOMe (25 wt % in MeOH, 1.4 mL, 6.12 mmol) was added. The reaction mixture was heated under reflux (78 °C) for 15 min, then cooled to room temperature and concentrated to half its original volume. Water (50 mL) was then added, affording a yellow precipitate. The suspension was washed with CH_2Cl_2 (2 × 20 mL), and the combined organic phases were washed with H_2O (2 × 20 mL), and brine (2 × 20 mL) then dried ($MgSO_4$). After filtration and removal of the solvent the compound was purified by column chromatography (deactivated SiO_2, CH_2Cl_2), to afford **4c'** as a yellow powder (0.25 g, 1.02 mmol, 89 %), mp 190 – 191 °C (lit. [17] 190 – 191 °C). ^{1}H NMR (500 MHz, $CDCl_3$): δ 11.08 (s, 1H), 6.78 (d, J = 2.7 Hz, 2H), 6.72 (s, 2H), ^{13}C NMR (125 MHz, $CDCl_3$): δ 109.7, 115.27, 116.2, 118.7, 120.0; MS-EI$^+$ m/z: 243; IR (KBr) ν/cm^{-1}: 3410, 3124, 1629, 1531, 1252; Anal. Calcd For $C_6H_5NS_4$ + 1/3 –CH_2–: C, 40.35; H, 2.30; N, 5.65. Found: C, 40.18; H, 2.11; N, 5.44.

2-(4,5-Bis(propylthio)-(1,3)-dithiole-2-ylidene)-(1,3)-dithiolo[4,5-*c*]pyrrole (4d'):

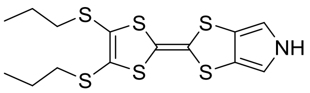

4d'

Compound **4d** (0.80 g, 1.47 mmol) was dissolved in a mixture of anhydrous THF (150 mL) and anhydrous MeOH (50 mL). The solution was degassed (Ar, 20 min) then placed under an argon atmosphere before NaOMe (25 wt % in MeOH, 0.55 mL, 2.41 mmol) was added. The reaction mixture was heated to reflux for 40 min then cooled to room temperature. Water (200 mL) was added and the mixture was washed with CH_2Cl_2 (2 × 100 mL). The combined organic phases were dried ($MgSO_4$). After filtration and removal of the solvent the compound was purified by column chromatography (deactivated SiO_2, CH_2Cl_2/petroleum ether 2:1 – 1:1), to afford compound **4d'** as orange crystals (0.54 g, 1.38 mmol, 94%), mp 88.3 – 89.7 °C (lit. [18] 87.5 – 88.0 °C). ^{1}H NMR (400 MHz, $CDCl_3$): δ 8.17 (bs, 1H), 6.61 (d, J = 2.7 Hz, 2H), 2.80 (t, J = 7.3 Hz, 4H), 1.67 (sextet, J = 7.3 Hz, 4H), 1.01 (t, J = 7.3 Hz, 6H); ^{13}C NMR (100 MHz, $CDCl_3$): δ 127.6, 120.2, 120.1, 111.4, 109.9, 38.4, 23.3, 13.3; MS-MALDI *m*/*z*: 391 (M^+, 100); IR (KBr) ν/cm^{-1}: 3348, 3123, 2955, 2919, 2867, 1452, 1374, 1049; Anal. Calcd for $C_{14}H_{17}NS_6$: C, 42.93; H, 4.37; N, 3.58; S, 49.12. Found: C, 43.08; H, 4.28; N, 3.58: S, 48.97.

2-((1,3)-Dithiole-2-ylidene)-*N*-(4-*tert*-butylthiophenyl)-(1,3)-dithiolo[4,5-*c*]pyrrole (4m):

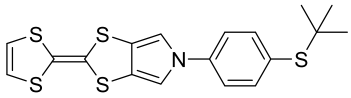

4m

Method A:

Compounds **4c'** (76 mg, 0.31 mmol) and **23**-Br (380 mg, 1.55 mmol), K_3PO_4 (293 mg, 1.30 mmol) and CuI (117 mg, 0.61 mmol) were placed in a Schlenk tube and dissolved in anhydrous THF (3.5 mL). The mixture was degassed (Ar, 15 min) then placed under an atmosphere of Ar before (±)-*trans*-1,2-diaminocyclohexane (0.1 mL, 0.83 mmol) was added and the Schlenk tube was sealed. The reaction mixture was heated for 14 h at 100 °C then cooled to room temperature. The solids were removed by filtration and washed with CH_2Cl_2 (2 × 20 mL). The filtrate was washed with NaOH (1 wt % in H_2O, 2 × 10 mL) and H_2O (2 × 10 mL) before the combined organic phases were dried ($MgSO_4$). After filtration and removal of the solvent the compound was purified by column chromatography (deactivated SiO_2, CH_2Cl_2/petroleum ether 1:2 – 1:1), to afford **4m** as a yellow solid (44 mg, 0.11 mmol, 35%). Characterisation was in agreement with the data given for Method B below.

Method B:

Compound **4c'** (76 mg, 0.31 mmol), CuI (106 mg, 0.54 mmol) and K_3PO_4 (305 mg, 1.35 mmol) were placed in a microwave reaction tube and dissolved in anhydrous THF (5 mL). The mixture was degassed (Ar, 15 min) then placed under an atmosphere of Ar, before a degassed solution of **23**-Br (170 mg, 0.69 mmol) and (±)-*trans*-1,2-diaminocyclohexane (0.04 mL, 0.33 mmol) in anhydrous THF (3 mL) was added via cannula. The tube was capped and the reaction was heated at 100 °C for 3 h in a microwave reactor then cooled to room temperature. Dichloromethane

(20 mL) was added to the mixture which was then washed with NaOH (1 wt % in H_2O, 2 × 20 mL), H_2O (3 × 15 mL) and brine (3 × 15 mL) before the organic phase was dried ($MgSO_4$). After filtration and removal of the solvent the compound was purified by column chromatography (deactivated SiO_2, CH_2Cl_2:petroleum ether 1:2 – 1:1), to afford **4m** as a yellow solid (104 mg, 0.25 mmol, 81%), mp 224 – 225 °C (dec). ^{1}H NMR(500 MHz, $(CD_3)_2SO$): δ 7.55 (s, 4H), 7.51 (d, J = 2.7 Hz, 2H), 6.76 (s, 2H), 1.25 (s, 9H); ^{13}C NMR (125 MHz, $(CD_3)_2SO$): δ 139.8, 138.4, 128.6, 121.9, 119.6, 118.8, 115.9, 112.9, 111.2, 45.9, 30.6; MS-MALDI *m/z*: 406.9861 (M^+, 100), 350.03 ([M – *t*Bu]$^+$, 24); IR (ATR) ν/cm^{-1}: 3138, 2958, 2921, 2360, 1590, 1505, 1380, 1314, 1167, 1032; Anal. Calcd for $C_{18}H_{17}NS_5$: C, 53.03; H, 4.20; N, 3.44. Found: C, 52.44; H, 4.17; N, 3.32.

2-(4,5-Bis(propylthio)-(1,3)-dithiole-2-ylidene)-*N*-(4-*tert*-butylthiophenyl)-(1,3)-dithiolo[4,5-*c*]pyrrole (4n):

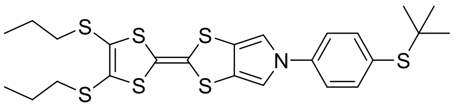

4n

Method A:

Compounds **4d'** (101 mg, 0.26 mmol) and **23**-Br (320 mg, 1.30 mmol), K_3PO_4 (219 mg, 1.03 mmol) and CuI (50 mg, 0.26 mmol) were placed in a sealable reaction vial under Ar and dissolved in degassed anhydrous THF (11 mL). The mixture was further degassed (Ar, 10 min) before (±)-*trans*-1,2-diaminocyclohexane (0.05 mL, 48 mg, 0.42 mmol) was added and the vial was sealed. The reaction mixture was heated for 48 h at 80 °C then cooled to room temperature. Dichloromethane (50 mL) was added to the mixture which was then washed with an aqueous solution of NaOH (0.25 M, 2 × 150 mL) and H_2O (2 × 150 mL). The aqueous phases were extracted

with CH_2Cl_2 (2 × 50 mL) and the organic phases combined with that from the previous extraction and dried ($MgSO_4$). After filtration and removal of the solvent the compound was purified by repeated column chromatography (deactivated SiO_2, CH_2Cl_2/cyclohexane 1:3) to afford a yellow solid which was recrystallised (CH_2Cl_2/cold MeOH) to afford **4n** as a yellow solid (98 mg, 0.18 mmol, 68%). Characterisation was in agreement with the data given for Method B below.

Method B:

Compounds **4d'** (90 mg, 0.22 mmol) and **23**-Br (170 mg, 0.69 mmol), CuI (78 mg, 0.54 mmol) and K_3PO_4 (173 mg, 0.81 mmol) were placed in a microwave reaction tube and dissolved in anhydrous THF (5 mL). The mixture was degassed (Ar, 15 min) then placed under Ar, before (±)-*trans*-1,2-diaminocyclohexane (0.07 mL, 0.06 g, 0.58 mmol) was added. The tube was capped and the reaction mixture was heated at 115 °C for 3 h in a microwave reactor then cooled to room temperature. Dichloromethane (20 mL) was added to the mixture which was then washed with NaOH (5 wt %in H_2O, 2 × 20 mL) and H_2O (3 × 15 mL) before the organic phase was dried ($MgSO_4$). After filtration and removal of the solvent the compound was purified by column chromatography (SiO_2, CH_2Cl_2/petroleum ether 1:2 – 1:1) to afford **4n** as a yellow solid (104 mg, 0.19 mmol, 93%), mp 92 – 94 °C. ^{1}H NMR (400 MHz, $(CD_3)_2SO$): δ 7.56 (s, 4H), 7.53 (s, 2H), 2.86 (t, J = 7.2 Hz, 4H), 1.59 (sextet, J = 7.2 Hz, 4H), 1.25 (bs, 9H), 0.97 (t, J = 7.2 Hz, 6H); ^{13}C NMR (125 MHz, $(CD_3)_2SO$): δ 139.6, 138.3, 128.7, 126.6, 121.1, 119.1, 118.8, 111.4, 109.6, 45.8, 37.5, 30.5, 22.5, 12.7; MS-MALDI m/z: 555.1176 (M^+, 100); IR (ATR) ν/cm^{-1}: 2956, 2918, 2869, 1590, 1503, 1454, 1310, 1166, 1034; Anal. Calcd for $C_{24}H_{29}NS_7$: C, 51.85; H, 5.26; N, 2.52; S, 40.37. Found: C, 51.90; H, 5.23; N, 2.51; S, 40.08.

2-(4,5-Bis(butylthio)-(1,3)-dithiole-2-ylidene)-*N*-(4-(trimethylsilylacetylenyl)phenyl)-(1,3)-dithiolo[4,5-*c*]pyrrole (4o):

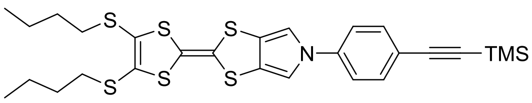

4o

Method A:

Compounds **4f'** (116 mg, 0.28 mmol) and **24**-I (518 mg, 1.70 mmol), CuI (112 mg, 0.59 mmol) and K_3PO_4 (216 mg, 1.02 mmol) were placed in a microwave reaction tube and dissolved in anhydrous THF (5 mL). The mixture was degassed (Ar, 10 min) then placed under Ar, before (±)-*trans*-1,2-diaminocyclohexane (0.14 mL, 133 mg, 1.17 mmol) was added. The tube was capped and the reaction mixture was heated at 118 °C for 3 h in a microwave reactor then cooled to room temperature. Dichloromethane (25 mL) was added to the mixture which was then washed with NaOH (1 wt % in H_2O, 2 × 15 mL) and H_2O (2 × 15 mL) before the organic phase was dried ($MgSO_4$). After filtration and removal of the solvent the compound was purified by column chromatography (SiO_2, CH_2Cl_2/petroleum ether 1:3), to afford **4o** as a yellow solid (89 mg, 0.150 mmol, 54%). Characterisation was in agreement with the data given for Method B below.

Method B:

Compounds **4f'** (1.05 g, 2.50 mmol) and **24**-I (3.60 g, 11.99 mmol), CuI (0.93 g, 4.88 mmol) and K_3PO_4 (1.78 g, 8.39 mmol) were dissolved in anhydrous THF (50 mL). The mixture was degassed (Ar, 20 min) then placed under Ar, before (±)-*trans*-1,2-diaminocyclohexane (1.2 mL, 1.14 g, 9.99 mmol) was added. The reaction mixture was heated to reflux under Ar for 3 h then cooled to room temperature. Dichloromethane (100 mL) was added to the mixture which was then washed with NaOH solution (1 wt % in H_2O; 3 × 150 mL) and H_2O (3 × 150 mL), before the

organic phase was dried ($MgSO_4$). After filtration and removal of the solvent the compound was purified by column chromatography (SiO_2, CH_2Cl_2/petroleum ether 1:3 – 1:1) to afford **4o** as yellow crystals (0.99 g, 1.67 mmol, 67%), mp 116.0 – 118.0 °C. ^1H NMR (500 MHz, CD_2Cl_2): δ 7.50 ppm (d, J = 8.8 Hz, 2H), 7.28 (d, J = 8.8 Hz, 2H), 6.94 (s, 2 H), 2.84 (t, J = 7.3 Hz, 4 H), 1.59–1.65 (m, 4H), 1.40–1.48 (m, 4H), 0.92 (t, J = 7.3 Hz, 6H), 0.25 (s, 9H); ^{13}C NMR (125 MHz, CD_2Cl_2): δ 140.3, 133.9, 128.2, 123.3, 121.2, 119.9, 111.2, 104.4, 95.6, 36.5, 32.4, 22.2, 13.9, 0.1; HRMS (EI) calcd for $C_{27}H_{33}NS_6Si^+$: 591.0701. Found: 591.1000; Anal. Calcd for $C_{27}H_{33}NS_6Si$: C, 54.78; H, 5.62; N, 2.37. Found: C, 54.97; H, 5.80; N, 2.38.

2-((1,3)-Dithiole-2-ylidene)-*N*-(4-formylphenyl)-(1,3)-dithiolo[4,5-*c*]pyrrole (4p):

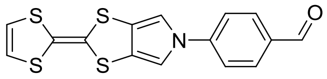

4p

Compounds **4c'** (59 mg, 0.25 mmol) and **25**-I (278 mg, 1.20 mmol), K_3PO_4 (178 mg, 0.84 mmol) and CuI (91 mg, 0.48 mmol) were placed in a Schlenk tube and dissolved in anhydrous THF (5 mL). The mixture was degassed (Ar, 15 min) then placed under an atmosphere of Ar before (±)-*trans*-1,2-diaminocyclohexane (0.1 mL, 95 mg, 0.83 mmol) was added and the Schlenk tube was sealed. The reaction mixture was heated for 3 h at 105 °C then cooled to room temperature. CH_2Cl_2 (20 mL) was added to the mixture, which was then washed with H_2O (4 × 20 mL) before the organic phase was dried ($MgSO_4$). After filtration and removal of the solvent the compound was purified by column chromatography (deactivated SiO_2, CH_2Cl_2:petroleum ether 1:2 – 1:1) to afford an orange solid, which was recrystallised from CH_2CH_2/Et_2O 1:1 to afford **4p** as orange crystals (30 mg, 0.09 mmol, 35 %), mp > 230 °C. ^1H NMR (500 MHz, $(CD_3)_2SO$): δ 9.98 (s, 1 H), 7.94 (d, J = 8.2 Hz, 2H),

7.45 (d, J = 8.2 Hz, 2H), 6.99 (s, 2H), 6.34 (s, 2H); ^{13}C NMR (125 MHz, $(CD_3)_2SO$): δ 190.8, 136.2, 131.7, 126.6, 119.7, 119.0, 118.6, 110.1; MS-MALDI *m/z:* 348 (M^+, 100); IR (ATR) ν/cm^{-1}: 3141, 3063, 2746, 2361, 1678, 1596, 1516, 1429, 1388, 1307, 1189, 1029; Anal. Calcd for $C_{15}H_9NOS_4$: C, 51.85; H, 2.61; N, 4.03. Found: C, 51.33; H, 2.49; N, 3.65.

2-((1,3)-Dithiole-2-ylidene)-*N*-((4-(2-cyanoethylthio)phenyl)-(1,3)-dithiolo[4,5-*c*]pyrrole (4q):

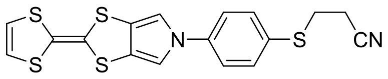

4q

Compounds **4c'** (53 mg, 0. 22 mmol) and **26**-I (315 mg, 1.08 mmol), K_3PO_4 (168 mg, 0.79 mmol) and CuI (82 mg, 0.43 mmol) were placed in a Schlenk tube and dissolved in anhydrous THF (5 mL) using an ultrasonic bath. The mixture was degassed (Ar, 10 min) then placed under an atmosphere of Ar before (±)-*trans*-1,2-diaminocyclohexane (0.1 mL, 95 mg, 0.83 mmol) was added and the Schlenk tube was sealed. The reaction mixture was heated for 3 h at 115 °C then cooled to room temperature. The solids were removed by filtration and washed with CH_2Cl_2 (2 × 20 mL). The filtrate was washed with NaOH (1 wt % in H_2O, 2 × 20 mL) and H_2O (2 × 20 mL) before the organic phase was dried ($MgSO_4$). After filtration and removal of the solvent the compound was purified by column chromatography (SiO_2, CH_2Cl_2/petroleum ether 1:2 – 1:1) to afford **4q** as a yellow solid (46 mg, 0.11 mmol, 52%) containing traces of grease, mp 222 – 224°C (dec). ^{1}H NMR (400 MHz, CD_3CN): δ 7.50 (d, J = 8.2 Hz, 2H), 7.40 (d, J = 8.2 Hz, 2H), 7.10 (s, 2H), 6.49 (s,

2H), 3.18 (t, J = 6.8 Hz, 2H), 2.67 (t, J = 6.8 Hz, 2H);[2] MS-MALDI m/z: 403.9858; IR (KBr) v/cm^{-1}: 2954, 2852, 2361, 2247, 1742, 1592, 1504, 1378, 1315, 1036. Anal. Calcd for $C_{17}H_{12}N_2S_5$ + ½ –CH_2–: C, 51.07; H, 3.18; N, 6.81. Found: C, 50.72; H, 3.67; N, 6.32.

2-(4,5-Bis(propylthio)-(1,3)-dithiole-2-ylidene)-*N*-(4-(2-cyanoethylthio)phenyl)-(1,3)-dithiolo[4,5-*c*]pyrrole (4r):

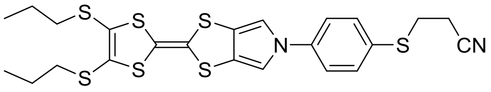

4r

Compounds **4d'** (40 mg, 0.10 mmol) and **26**-I (147 mg, 0.50 mmol), K_3PO_4 (89 mg, 0.42 mmol) and CuI (48 mg, 0.25 mmol) were placed in a Schlenk tube and dissolved in anhydrous THF (5 mL) using an ultrasonic bath. The mixture was degassed (Ar, 10 min) then placed under an atmosphere of Ar before (±)-*trans*-1,2-diaminocyclohexane (0.05 mL, 48 mg, 0.42 mmol) was added and the Schlenk tube was sealed. The reaction mixture was heated for 3 h at 115 °C then cooled to room temperature. The solids were removed by filtration and CH_2Cl_2 (20 mL) was added to the filtrate, which was washed with NaOH (1 wt % in H_2O, 2 × 20 mL), H_2O (2 × 20 mL) and brine (2 × 20 mL) before the organic phase was dried ($MgSO_4$). After filtration and removal of the solvent the compound was purified by column chromatography (SiO_2, CH_2Cl_2/petroleum ether 1:2 – 1:1) to afford a yellow solid which was recrystallized from CH_2Cl_2/ Et_2O 1:1 to afford **4r** as a yellow solid (36 mg, 0.07 mmol, 65%), mp 106 – 109 °C. ^{1}H NMR (500 MHz, $CDCl_3$): δ 7.49 (d, J = 8.6 Hz, 2H), 7.34 (d, J = 8.6 Hz, 2H), 6.88 (s, 2H), 3.12 (t, J = 7.2 Hz, 2H), 2.81 (t, J = 7.2 Hz, 4H), 2.61 (t, J = 7.2 Hz, 2H), 1.68 (q, J = 7.2 Hz, 4H), 1.02 (t, J = 7.3 Hz, 6H);

[2] It was not possible to obtain a ^{13}C NMR spectrum for this compound as it rapidly precipitated from all suitable solvents.

^{13}C NMR (125 MHz, $CDCl_3$): δ 133.6, 132.2, 130.9, 123.2, 117.9, 38.4, 31.0, 23.3, 18.5, 13.3;[3] MS-MALDI *m*/*z*: 553.08 (M^+ , 100).

2-(4,5-Bis(methylthio)-(1,3)-dithiole-2-ylidene)-*N*-(4-*tert*-butylthiophenyl)-4,6-dimethyl-(1,3)-dithiolo[4,5-*c*]pyrrole (4s):

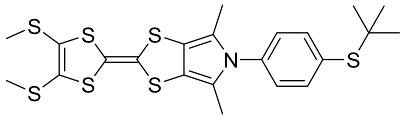

4s

Compound **4g'** (60 mg, 0.166 mmol), CuI (55 mg, 0.29 mmol) and K_3PO_4 (174 mg, 0.82 mmol) were placed in a Schlenk tube under an atmosphere of Ar before a degassed solution of **23**-Br (0.16 mL, 0.65 mmol) and (±)-*trans*-1,2-diaminocyclohexane (0.07 mL, 67 mg, 0.58 mmol) in anhydrous THF (5 mL) was added via cannula and the Schlenk tube was sealed. The reaction mixture was heated for 24 h at 90 °C then cooled to room temperature. Dichloromethane (20 mL) was added to the mixture, which was washed with NaOH (1 wt % in H_2O, 3 × 10 mL) and H_2O (3 × 20 mL) before the organic phase was dried ($MgSO_4$). After filtration and removal of the solvent the compound was purified by column chromatography (SiO_2, CH_2Cl_2/petroleum ether 1:2 – 1:1) to afford **4s** as an orange-yellow solid (22 mg, 0.04 mmol, 25%), mp 177–180 °C. ^1H NMR (400 MHz, $(CD_3)_2SO$): δ 7.63 (d, J = 8.4 Hz, 2H), 7.36 (d, J = 8.4 Hz, 2H), 2.45 (s, 6H), 1.96 (s, 6H), 1.29 (s, 9H); ^{13}C NMR (100 MHz, $CDCl_3$): δ 138.9, 138.5, 135.9, 133.1, 131.4, 116.5, 46.3, 31.5, 12.9;[3] MS-MALDI *m*/*z*: 527.09 (M^+); IR (KBr) ν/cm^{-1}: 3054, 2916, 2361, 2340, 1587, 1469, 1455, 1386, 1373, 1361, 1315, 1264, 1163, 1092, 1015.

[3] Some signals are missing or overlapping.

2-(4,5-Bis(propylthio)-(1,3)-dithiole-2-ylidene)-*N*-(4-(trimethylsilyl-acetylenyl)phenyl)-(1,3)-dithiolo[4,5-*c*]pyrrole (4u):

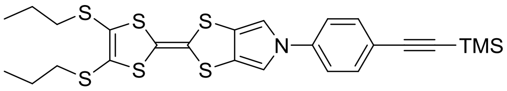

4u

Compounds **4d'** (212 mg, 0.54 mmol) and **26**-I (805 mg, 2.68 mmol), CuI (100 mg, 0.54 mmol) and K_3PO_4 (458 mg, 2.16 mmol) were placed in a microwave reaction tube under an atmosphere of Ar then dissolved in degassed anhydrous THF (18 mL). The mixture was further degassed (Ar, 15 min) before (±)-*trans*-1,2-diaminocyclohexane (0.11 mL, 105 mg, 0.92 mmol) was added. The tube was capped and the reaction mixture was heated at 118 °C for 2 h in a microwave reactor then cooled to room temperature. Dichloromethane (100 mL) was added to the mixture which was then washed with an aqueous solution of NaOH (0.25 M, 2 × 200 mL) and H_2O (2 × 200 mL). The combined aqueous phases were extracted with CH_2Cl_2 (100 mL) and the organic phase combined with that from the previous extraction and dried ($MgSO_4$). After filtration and removal of the solvent the compound was purified by repeated column chromatography (deactivated SiO_2, CH_2Cl_2/cyclohexane 1:3) to afford **4u** as a yellow solid (156 mg, 0.28 mmol, 51%), mp 122.8 °C. ^{1}H NMR (500 MHz, $CDCl_3$): δ 7.53 – 7.46 (m, 2H), 7.25 – 7.20 (m, 2H), 6.89 (s, 2H), 2.81 (t, J = 7.3 Hz, 4H), 1.67 (sextet, J = 7.3 Hz, 4H), 1.02 (t, J = 7.3 Hz, 6H), 0.25 (br. S, 9H); ^{13}C NMR (100 MHz, $CDCl_3$): δ 139.9, 133.6, 127.7, 123.2, 120.8, 119.3, 118.7, 112.6, 110.6, 104.2, 95.3, 38.4, 23.3, 13.3, 0.1; MS-MALDI *m/z*: 562.58 (M^+); Anal. Calcd for $C_{25}H_{29}NS_6Si$: C, 53.24; H, 5.18; N, 2.48; S, 34.11. Found: C, 53.42; H, 5.10; N, 2.50; S, 34.24.

2-(4,5-Bis(propylthio)-(1,3)-dithiole-2-ylidene)-*N*-(4-aminophenyl)-(1,3)-dithiolo[4,5-*c*]pyrrole (4v):

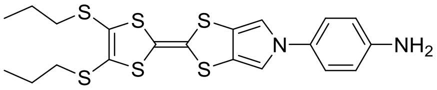

4v

Compounds **4d'** (206 mg, 0.53 mmol) and **27**-I (306 mg, 1.40 mmol), CuI (200 mg, 1.05 mmol), K_3PO_4 (416 mg, 1.96 mmol) and (±)- *trans*-1,2-diaminocyclohexane (0.1 mL, 95 mg, 0.83 mmol) were placed in a microwave reaction tube and dissolved in anhydrous THF (6 mL) using an ultrasonic bath. The mixture was degassed (Ar, 20 min) then placed under Ar. The tube was capped and the reaction mixture was heated at 130 °C for 2 h in a microwave reactor then cooled to room temperature. The mixture was poured into CH_2Cl_2 (100 mL) which was then washed with NaOH (5 wt % in H_2O, 100 mL) and H_2O (2 × 100 mL) before the organic phase was dried ($MgSO_4$). After filtration and removal of the solvent the compound was purified by column chromatography (SiO_2, CH_2Cl_2/petroleum ether 3:2) to afford **4v** as a brown solid (204 mg, 0.42 mmol, 80%), mp 115 – 117 °C. ^{1}H NMR (400 MHz, $CDCl_3$): δ 7.09 (d, J = 8.6 Hz, 2H), 6.75 (s, 2H), 6.70 (d, J = 8.6 Hz, 2H), 3.72 (s, 2H), 2.81 (t, J = 7.3 Hz, 4H), 1.67 (sextet, J = 7.3 Hz, 4H), 1.02 (t, J = 7.3 Hz, 6H); ^{13}C NMR (100 MHz, $CDCl_3$): δ 145.2, 127.6, 122.4, 121.0, 115.8, 111.6, 38.4, 23.3, 13.3;[4] HRMS (FT-ESI) calcd for $C_{20}H_{22}N_2S_6^{+\bullet}$ 482.0107. Found 482.0056.

[4] Some signals are missing or overlapping.

2-(4,5-Bis(propylthio)-(1,3)-dithiole-2-ylidene)-*N*-(4-cyanophenyl)-(1,3)-dithiolo[4,5-*c*]pyrrole (4w):

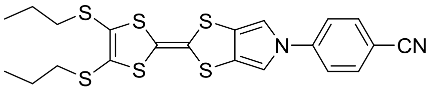

4w

Compounds **4d'** (75 mg, 0.19 mmol) and **28**-I (211 mg, 0.92 mmol), CuI (77 mg, 0.40 mmol) and K_3PO_4 (161 mg, 0.76 mmol) were placed in a microwave reaction tube and dissolved in anhydrous THF (4 mL). The mixture was degassed (Ar, 15 min) then placed under an atmosphere of Ar, before (±)-*trans*-1,2-diaminocyclohexane (0.07 mL, 67 mg, 0.58 mmol) was added. The tube was capped and the reaction mixture was heated at 115 °C for 3 h in a microwave reactor then cooled to room temperature. Dichloromethane (20 mL) was added to the mixture which was then washed with NaOH (5 wt % in H_2O, 2 × 20 mL) and H_2O (2 × 20 mL) before the organic phase was dried ($MgSO_4$). After filtration and removal of the solvent the compound was purified by column chromatography (SiO_2, CH_2Cl_2/petroleum ether 1:2) to afford **4w** as a shiny yellow solid (60 mg, 0.12 mmol, 64%), mp > 230 °C. ^{1}H NMR (400 MHz, $(CD_3)_2SO$): δ 7.94 (d, J = 8.7 Hz, 2H), 7.74 (d, J= 8.7 Hz, 2H), 7.63 (s, 2H), 2.86 (t, J = 7.2 Hz, 4H), 1.59 (sextet, J = 7.2 Hz, 4H), 0.97 (t, J = 7.2 Hz, 6H); ^{13}C NMR (125 MHz, CS_2: $(CD_3)_2SO$): δ 141.6, 133.0, 127.2, 125.0, 118.4, 117.4, 116.3, 113.0, 109.3, 109.2, 38.0, 23.1, 13.1; MS-MALDI *m*/*z*: 492.003; IR (ATR): ν/cm^{-1}: 3135, 2964, 2924, 2873, 2221, 1603, 1518, 1382, 1314, 1185, 1029; Anal. Calcd for $C_{21}H_{20}N_2S_6$: C, 51.18; H, 4.09; N, 5.68; S, 39.04. Found: C, 51.27; H, 4.05; N, 5.62; S, 39.19.

Bis(pyrrolo[3,4-*d*])tetrathiafulvalene (5a'):

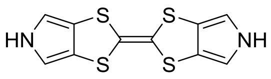

5a'

Compound **5a** (500 mg, 0.85 mmol) was dissolved in mixture of anhydrous THF (35 mL) and anhydrous MeOH (35 mL). The solution was degassed (Ar, 15 min) then placed under an atmosphere of Ar before NaOMe (25 wt % in MeOH, 2 mL, 473 mg, 8.75 mmol) was added. The mixture was heated under reflux for 30 min, then cooled to room temperature and concentrated to half its original volume. Water (100 mL) was then added, affording a yellow precipitate, which was isolated by filtration and washed with H_2O (2 × 20 mL) Purification of the precipitate by column chromatography (deactivated Al_2O_3, THF/MeOH 50:1) afforded **5a'** as a yellow solid (237 mg, 0.84 mmol, 99%), mp > 236 °C (dec.) (lit. [12] dec. 215 – 220 °C). ^{1}H NMR (400 MHz, $(CD_3)_2SO$): δ 11.08 (2H, bs), 6.78 (d, J = 2.4 Hz, 4H); ^{13}C NMR (100 MHz, $(CD_3)_2SO$): δ 119.3, 117.0, 110.3; MS-MALDI *m/z*: 281.94 (M^+, 21), 185.11 (100); IR (KBr) v/cm^{-1}: 3127, 3117; Anal. Calcd for $C_{10}H_6N_2S_4$: C, 42.53; H, 2.14; N, 9.92. Found: C, 42.14; H, 2.08; N, 9.50.

Bis(*N*-(4-*tert*-butylthiophenyl)pyrrolo[3,4-*d*])tetrathiafulvalene (5c):

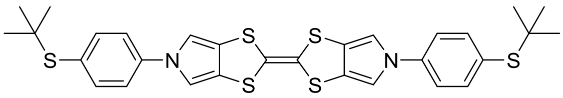

5c

Method A:

Compound **5a'** (90 mg, 0.22 mmol), CuI (78 mg, 0.54 mmol) and K_3PO_4 (173 mg, 0.81 mmol) were placed in a Schlenk tube under Ar before a degassed solution of **23**-Br (170 mg, 0.69 mmol) and (±)-*trans*-1,2-diaminocyclohexane (0.07 mL, 67 mg, 0.58 mmol) in anhydrous THF (5 mL) was added via cannula and the Schlenk tube

was sealed. The reaction mixture was heated for 24 h at 93 °C then cooled to room temperature. The resulting yellow precipitate was collected by filtration and dissolved in a minimum of CH_2Cl_2 (ca. 10 mL). This solution was washed with NaOH (5 wt % in H_2O, 3 × 10 mL), H_2O (3 × 10 mL) and brine (3 × 20 mL) before the organic phase was dried (Na_2SO_4). Filtration and removal of the solvent afforded compound **5c** as a yellow solid (57 mg, 0.09 mmol, 42%). Characterisation was in agreement with the data given for Method B below.

Method B:

Compounds **5a'** (134 mg, 0.47 mmol) and **23**-Br (580 mg, 2.37 mmol), CuI (180 mg, 0.95 mmol) and K_3PO_4 (408 mg, 1.92 mmol) were placed in a Schlenk tube under N_2 then dissolved in degassed 1,4-dioxane (6 mL). (±)-*trans*-1,2-Diaminocyclohexane (0.21 mL, 200 mg, 1.75 mmol) was then added and the Schlenk tube was sealed. The reaction mixture was heated for 40 h at 110 °C then cooled to room temperature. The reaction mixture was treated with CH_2Cl_2 (10 mL), affording a yellow suspension, which was carefully washed with NaOH (1 wt % in H_2O, 3 × 10 mL), H_2O (3 × 10 mL) and brine (3 × 20 mL). The organic phase was filtered, the obtained solid washed with CH_2Cl_2 and dried in vacuo to afford **5c** as a yellow solid[5] (210 mg, 0.34 mmol, 73%), mp > 250 °C. MS-EI$^+$ *m/z*: 610 (M^+); Anal. Calcd for $C_{30}H_{30}N_2S_6$: C, 58.98; H, 4.95; N, 4.59; S, 31.49. Found: C, 58.45; H, 4.75; N, 4.54; S, 31.17.

[5] No further purification was carried out due to the poor solubility of the compound. Similarly, it was not possible to obtain ^1H or ^{13}C NMR spectra.

Bis(*N*-(4-(2-cyanoethylthio)phenyl)pyrrolo[3,4-*d*])tetrathiafulvalene (5e):

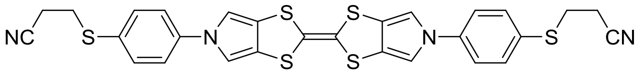

5e

Compounds **5a'** (40 mg, 0.14 mmol) and **26**-I (0.205 mg, 0.71 mmol), CuI (53 mg, 0.28 mmol) and K_3PO_4 (110 mg, 0.52 mmol) were dissolved in anhydrous THF (5 mL). The mixture was degassed (Ar, 15 min) then placed under an atmosphere of Ar before (±)-*trans*-1,2-diaminocyclohexane (0.07 mL, 67 mg, 0.58 mmol) was added and the Schlenk tube was sealed. The reaction mixture was heated for 3 h at 115 °C then cooled to room temperature. The resulting yellow precipitate was collected by filtration and dissolved in a minimum of CH_2Cl_2 (ca. 10 mL). This solution was washed with NaOH (5 wt % in H_2O, 3 × 10 mL), H_2O (3 × 10 mL) and brine (3 × 20 mL) before the organic phase was dried (Na_2SO_4). After filtration and removal of the solvent the compound was purified by column chromatography (SiO_2, CH_2Cl_2/petroleum ether 1:2 – 1:1) to afford **5e** as a yellow solid (21 mg, 0.03 mmol, 24%); mp > 250°C. ^{1}H NMR (300 MHz, $CDCl_3$): δ 7.61 (d, J = 8.2 Hz, 4H), 7.22 (d, J = 8.2 Hz, 4H), 7.09 (d, J = 7.6 Hz, 4H), 3.06 (t, J = 7.2 Hz, 4H), 2.54 (t, J = 7.2 Hz, 4H); MS-MALDI *m*/*z*: 604.2511 (M^+, 100); IR (KBr) ν/cm^{-1}: 3436, 2929, 2857, 2249, 1661, 1641, 1591, 1504, 1448, 1348, 1315,1100, 1036, 820. The difficulties encountered in fully purifying this poorly soluble compound meant a satisfactory elemental analysis was not obtained.

Bis-(*N*-(4-*tert*-butylthiophenyl)-2,5-dimethylpyrrolo[3,4-*d*])tetrathiafulvalene (5f):

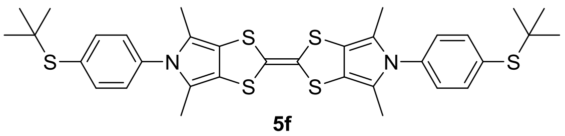

5f

Method A:

Compound **5b'** (81 mg, 0.24 mmol), CuI (92 mg, 0.48 mmol) and K_3PO_4 (181 mg, 0.85 mmol) were placed in a Schlenk tube under an atmosphere of Ar before a degassed solution of **23**-Br (320 mg, 1.10 mmol) and (±)-*trans*-1,2-diaminocyclohexane (0.11 mL, 105 mg, 0.92 mmol) in anhydrous THF (5 mL) was added via cannula and the Schlenk tube was sealed. The reaction mixture was heated for 48 h at 105 °C then cooled to room temperature. The resulting yellow precipitate was removed by filtration and dissolved in a minimum of CH_2Cl_2 (ca. 10 mL). This solution was washed with NaOH (5 wt % in H_2O, 3 × 10 mL), H_2O (3 × 10 mL) and brine (3 × 20 mL) before the organic phase was dried (Na_2SO_4). After filtration and removal of the solvent the compound was purified by column chromatography (deactivated SiO_2, CH_2Cl_2/petroleum ether 1:2 – 1:1) to afford **5f** as a yellow solid (64 mg, 0.10 mmol, 40%). Characterisation was in agreement with the data given for Method B below.

Method B:

Compounds **5b'** (75 mg, 0.22 mmol) and **23**-Br (500 mg, 2.04 mmol), CuI (85 mg, 0.45 mmol) and K_3PO_4 (190 mg, 0.89 mmol) were placed in a Schlenk tube under an atmosphere of N_2 then dissolved in degassed 1,4-dioxane (4 mL). (±)-*trans*-1,2-Diaminocyclohexane (0.1 mL, 95 mg, 0.83 mmol) was then added and the Schlenk tube was sealed. The reaction mixture was heated for 65 h at 110 °C then cooled to room temperature. The reaction mixture was treated with CH_2Cl_2 (10 mL) and the

solution was washed with NaOH (1 wt % in H_2O, 3 × 10 mL), H_2O (3 × 10 mL) and brine (3 × 20 mL) before the organic phase was dried ($MgSO_4$). After filtration and removal of the solvent the compound was purified by column chromatography (deactivated SiO_2, petroleum ether, followed by CH_2Cl_2/petroleum ether 1:1) to afford **5f** as a yellow solid (35 mg, 0.05 mmol, 24%), mp > 250 °C. ^1H NMR (400 MHz, $CDCl_3$): δ 7.62 (d, J = 7.8 Hz, 4H), 7.13 (d, J = 7.8 Hz, 4H), 1.99 (s, 12 H), 1.33 (s, 18 H); ^{13}C NMR (100 MHz, $CDCl_3$): δ 138.4, 133.2, 116.7, 46.5, 31.2, 12.7; MS-ESI$^+$ *m*/*z*: 666.14 (M^+); HRMS (FT-ESI) calcd. for $C_{34}H_{38}N_2S_6^+$: 666.1354. Found: 666.1352; Anal. Calcd for $C_{34}H_{38}N_2S_6$: C, 61.22; H, 5.74; N, 4.20; S, 28.84. Found: C, 61.50; H, 5.86; N, 4.17; S, 28.58.

References

1. Mekelburger, H.-B.; Groβ, J.; Schmitz, J.; Nieger, M.; Vögtle, F. *Chem. Ber.* **1993**, *126*, 1713.

2. Zong, K.; Chen, W.; Cava, M. P.; Rogers, R. D. *J. Org. Chem.* **1996**, *61*, 8117.

3. Söderbäck, E.; Gronowitz, S.; Hörnfeldt, A.-B. *Acta Chem. Scand.* **1961**, *15*, 227.

4. Simonsen, K. B.; Svenstrup, N.; Lau, J.; Simonsen, O.; Mørk, P.; Kristensen, G. J.; Becher, J. *Synthesis* **1996**, *1996*, 407.

5. Marquis, D.; Desvergne, J.-P.; Bouas-Laurent, H. *J. Org. Chem.* **1995**, *60*, 7984.

6. Ikeda, T.; Higuchi, M. *Tetrahedron* **2011**, *67*, 3046.

7. Sorensen, A.; Andersen, S. S.; Flood, A. H.; Jeppesen, J. O. *Chem. Commun.* **2013**, *49*, 5936.

8. Stuhr-Hansen, N. *Synth. Commun.* **2003**, *33*, 641.

9. Haiss, W.; Wang, C.; Grace, I.; Batsanov, A. S.; Schiffrin, D. J.; Higgins, S. J.; Bryce, M. R.; Lambert, C. J.; Nichols, R. J. *Nat Mater* **2006**, *5*, 995.

10. Solano, M. V.; Nielsen, M. B.; Jeppesen, J. O. *J. Heterocycl. Chem.*, in press.

11. Salinas, Y.; Solano, M. V.; Sørensen, R. E.; Larsen, K. R.; Lycoops, J.; Jeppesen, J. O.; Martínez-Máñez, R.; Sancenón, F.; Marcos, M. D.; Amorós, P.; Guillem, C. *Chem. Eur. J.* **2014**, *20*, 855.
12. Jeppesen, J. O.; Takimiya, K.; Jensen, F.; Brimert, T.; Nielsen, K.; Thorup, N.; Becher, J. *J. Org. Chem.* **2000**, *65*, 5794.
13. Solano, M. V.; Della Pia, E. A.; Jevric, M.; Schubert, C.; Wang, X.; van der Pol, C.; Kadziola, A.; Nørgaard, K.; Guldi, D. M.; Nielsen, M. B.; Jeppesen, J. O. *Chem. Eur. J.* **2014**, *20*, 9918.
14. Melby, L. R.; Hartzler, H. D.; Sheppard, W. A. *J. Org. Chem.* **1974**, *39*, 2456.
15. Jeppesen, J. O.; Takimiya, K.; Jensen, F.; Brimert, T.; Nielsen, K.; Thorup, N.; Becher, J. *J. Org. Chem.* **2000**, *65*, 5794.
16. Choi, J. W.; Flood, A. H.; Steuerman, D. W.; Nygaard, S.; Braunschweig, A. B.; Moonen, N. N. P.; Laursen, B. W.; Luo, Y.; DeIonno, E.; Peters, A. J.; Jeppesen, J. O.; Xu, K.; Stoddart, J. F.; Heath, J. R. *Chem. Eur. J.* **2006**, *12*, 261.
17. Nygaard, S.; Hansen, C. N.; Jeppesen, J. O. *J. Org. Chem.* **2007**, *72*, 1617.
18. Hansen, J. A.; Becher, J.; Jeppesen, J. O.; Levillain, E.; Nielsen, M. B.; Petersen, B. M.; Petersen, J. C.; Sahin, Y. *J. Mater. Chem.* **2004**, *14*, 179.
